# Macro and trace mineral constituents and radionuclides in mushrooms: health benefits and risks

**DOI:** 10.1007/s00253-012-4552-8

**Published:** 2012-11-25

**Authors:** Jerzy Falandysz, Jan Borovička

**Affiliations:** 1 Institute of Environmental Sciences & Public Health, University of Gdańsk, Gdańsk, Poland; 2 Nuclear Physics Institute, v.v.i., Academy of Sciences of the Czech Republic, Řež 130, CZ-250 68 Řež near Prague, Czech Republic; 3 Institute of Geology, v.v.i., Academy of Sciences of the Czech Republic, Rozvojová 269, CZ-165 00 Prague 6, Czech Republic

**Keywords:** Environment, Food, Fungi, Organic food, Se bioenrichment, Wild food

## Abstract

This article reviews and updates data on macro and trace elements and radionuclides in edible wild-grown and cultivated mushrooms. A huge biodiversity of mushrooms and spread of certain species over different continents makes the study on their multi-element constituents highly challenging. A few edible mushrooms are widely cultivated and efforts are on to employ them (largely *Agaricus* spp., *Pleurotus* spp., and *Lentinula edodes*) in the production of selenium-enriched food (mushrooms) or nutraceuticals (by using mycelia) and less on species used by traditional medicine, e.g., *Ganoderma lucidum*. There are also attempts to enrich mushrooms with other elements than Se and a good example is enrichment with lithium. Since minerals of nutritional value are common constituents of mushrooms collected from natural habitats, the problem is however their co-occurrence with some hazardous elements including Cd, Pb, Hg, Ag, As, and radionuclides. Discussed is also the problem of erroneous data on mineral compounds determined in mushrooms.

**Electronic supplementary material:**

The online version of this article (doi:10.1007/s00253-012-4552-8) contains supplementary material, which is available to authorized users.

## Introduction

Mushrooms are heterotrophic eukaryotic organisms classified in the kingdom of Fungi. Recent estimates based on high-throughput sequencing methods suggest that there are as many as 5.1 million fungal species worldwide (Blackwell 2011). The European continent has witnessed the highest number of studies in this area, and in this continent, at least 75,000 species exist, and of these, more than 15,000 species are macrofungi, i.e., fungi forming fruit bodies (sporocarps) that are visible to the naked eye (Senn-Irlet et al. 2007). The fruit body is the morphological part of the fungus that bears spores and is commonly called mushroom. There are a variety of forms of mushrooms. Depending on the situation, context, and language, the term mushroom is used also as synonymous to any fungus. In the context of this article, the term mushroom relates to the fleshy fruit body of the macrofungi that can be edible or inedible regardless of its shape and kind. According to their lifestyle, mushrooms are recognized as saprotrophs, parasites, and mutualists—ectomycorrhizal symbionts (Gadd 2008).

The study of the role that fungi have played and are playing in geological processes is termed geomycology (Gadd 2007). The fundamental importance of fungi involves organic and inorganic transformations and element cycling, rock and mineral transformations, bioweathering, fungal–clay interactions, metal–fungal interactions, and mycogenic mineral formation. However, despite the crucial roles of fungi in biogeochemical cycling of elements, the extent to which fungi play a critical role in the Earth’s processes is not always appreciated (Hawksworth 2009).

Similar to plants and microbes, fungi are intimately involved in soil mineral weathering and element cycling in the critical zone (Amundson et al. 2007); their mycelia colonize both organic and mineral soils and produce a variety of active chemical compounds including enzymes (Baldrian 2008) and various organic acids (Stijve and de Meijer 1999; Wallander and Wickman 1999; van Schöll et al. 2006a).

Production of enzymes in saprotrophic macrofungi plays an important role in the transformation of humic substances including humus formation and mineralization (Baldrian 2008) and mobilization of metals associated with organic matter. Extracellular enzymes (1) allow access to organic matter in the microporous layer of soil; (2) transform soil organic matter, which is often insoluble or adsorbed on mineral surfaces, into soluble products; and (3) decrease the structural complexity of soil organic matter (Quiquampoix and Burns 2008). Through enzyme production, the ectomycorrhizal mushrooms can utilize organic N and P forms which would otherwise remain largely unavailable to roots (Landeweert et al. 2001). Nutrient mobilization from amino acids, peptides, proteins, amino sugars, chitin, and nucleic acids has been shown (Chalot and Brun 1998), together with transfer of N and P into the host plant (Antibus et al. 1997). Production of organic acids leads to chemical leaching of mineral surfaces and consequent mobilization of nutrients like P, K, Ca, and Mg that can be translocated to the host plants (Jentschke et al. 2000; Wallander 2000a, b; Wallander and Wickman 1999).

**Fig. 1:**
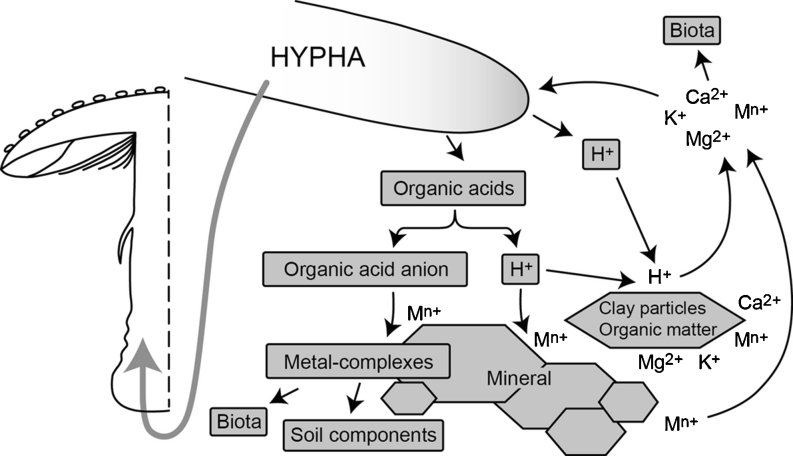
Proton- and organic acid ligand-mediated dissolution of metals of soil components/minerals and their transfer to fruit body (modified from Gadd 2004)

Analogous to their organic nutrient mobilizing capabilities, the abilities of different ectomycorrhizal fungi to mobilize inorganic nutrients might be species specific (van Schöll et al. 2006b; Plassard et al. 2011). In vitro experiments have demonstrated that mycelia of mushrooms can solubilize various types of metals and minerals (Fomina et al. 2006, 2007). On the other hand, this process was often accompanied by precipitation of various “mycogenic” minerals on fungal hyphae (Gadd et al. 2012; Rhee et al. 2012). The interactions between ectomycorrhizal fungi and metal elements have been recently reviewed by Urban (2011). The complex chemical action of macrofungi in soils described above leads, however, to a very interesting and not yet well-understood phenomenon: uptake, transport, and accumulation of various chemical elements in mushroom fruit bodies (Fig. 1).

Apart from hyphae (mycelium), rhizomorphs, which are root-like structures and which are known to be spread by species over a huge area of forest, also take part in the uptake of mineral elements. The contribution of rhizomorphs as a highly efficient medium for water and mineral compounds uptake from soils seems to explain the presence of Hg and Ag in fruiting bodies of *Armillaria solidipes* (Falandysz et al. 2012e; J.F., unpublished). An individual organism of *Armillaria bulbosa* could spread the mycelium and rhizomorphs over 150,000 m^2^ of forest (Smith et al. 1992).

## Historical and subject overview

The first reports on the chemical composition of macrofungal fruit bodies appeared more than 100 years ago (Zellner 1907) and covered several macroelements including Fe and Mn. Curious enough, Friese (1929) had reported strikingly high Fe concentrations in *Suillus variegatus* that were rediscovered 46 years later (Drbal et al. 1975a). However, reliable data appeared with the development of advanced instruments and validated analytical methods, especially after 1970 (Drbal et al. 1975a, b; Stijve and Besson 1976; Byrne et al. 1976; Bakken and Olsen 1990; Falandysz et al. 2001). Very high concentrations of toxic metallic elements (Hg, Cd), toxic metalloids (As), essential and toxic nonmetal (Se), and other elements (Ag, Au, Cs, Rb, V, Zn) found in mushrooms from “background areas” have led to many studies which are, however, mostly from European countries. These studies were focused especially on:

1. Toxic metallic elements and metalloids (As, Sb) which might represent a risk for mushroom consumers (see below)
2. Possibilities of using mushrooms for environmental monitoring (Wondratschek and Röder 1993)
3. Radioactive contamination before and after the Chernobyl accident (Reisinger 1994; see below)
4. Chemical form of accumulated elements in mushroom fruit bodies (Collin-Hansen et al. 2003; Šlejkovec et al. 1997, 2000)
5. Influence of environmental pollution on metallic elements accumulation (Kalač et al. 1991; Borovička et al. 2006, 2010b; Falandysz et al. 2012b; Kojta et al. 2012)
6. Chemotaxonomical and mycological systematics significance of trace element accumulation (Stijve et al. 1998, 2001; Petrini et al. 2009)
7. Mushroom cultivation on artificially metallic element-enriched substrates and media (Falandysz et al. 1994b; Crane et al. 2010)
8. Use of stable and radioactive isotopes for applications in the study of fungi (Rühm et al. 1997; Komárek et al. 2007; Guillén et al. 2009; Hobbie and Agerer 2010; Agerer et al. 2012).

The most recent studies usually considered various ecological/environmental aspects (Collin-Hansen et al. 2005; Falandysz et al. 2008a, 2012a; Borovička et al. 2010a; Vinichuk et al. 2011; Aloupi et al. 2011; Gryndler et al. 2012) or molecular approaches are applied to investigate metal transport and sequestration in macrofungi (Courbot et al. 2004; Ramesh et al. 2009; Blaudez and Chalot 2011; Osobová et al. 2011).

## Bioaccumulation in macrofungi

In contrast to vascular plants in which high concentrations of metals are mostly observed above metal-rich soils (Kabata-Pendias 2011), macrofungi can accumulate extremely high concentrations of metallic elements even when growing above soils with low metal contents. Furthermore, macrofungi reveal variable and highly specific ability to take up various trace elements (metallic elements, metalloids, and halogens) from soils and accumulate them in their fruit bodies. The ratio of an element in fruit body to its concentration in soil or, more specifically, to its mobility in soil (i.e., extractability by appropriate chemicals), so-called bioaccumulation factor (BAF), describes the ability of a fungus to accumulate a particular element. Apart from BAF, some other synonymous unit-less terms (transfer rate; transfer factor; transfer coefficient; translocation factor; concentration ratio; enrichment factor; index of bioaccumulatiuon, bioconcentration factor), which are simply a quotient from element concentration distributed between two phases, are also used by authors to estimate the potential of species to accumulate elements from soils or other substratum.

Elements typically accumulated in (at least some) macrofungi (BAF > 1) include Au, Ag, As, Br, Cd, Cl, Cs, Cu, Hg, Rb, Se, V, and Zn. Elements with typically low concentrations in macrofungi (BAF < 1) include Co, Cr, F, I, Ni, Sb, Sn, Th, U, and rare earth elements.

In some cases, concentrations of metallic element or metalloid might be rather high in some macrofungi (e.g., 10 or more times greater than in common ones, but BAF < 1); this phenomenon has been reported for Fe in *S. variegatus* and *Hygrophoropsis aurantiaca*, Co in *Agaricus* spp., Mn in *Phallaceae* and *Panaeolus* spp., Pb in *Lycoperdon*, and Sb in *Suillus* spp. and *Chalciporus piperatus* (Falandysz et al. 2001b; Borovička et al. 2006).

Factors influencing the bioaccumulation of trace elements in macrofungi and the biological importance of the accumulation process itself are poorly understood. However, the following fundamentals have been recognized:

1. Natural factors (bedrock geochemistry): The type of geological bedrock influences the concentrations of trace elements. Furthermore, pH/Eh conditions, organic matter content, and other factors influence the “mobility” of elements and their availability to biota (Kabata-Pendias 2011). The influence of these factors on trace element uptake in macrofungi has been poorly investigated. Gast et al. (1988) did not find any influence of soil pH or organic matter on Cd, Cu, Pb, and Zn in *Amanita muscaria*, *Amanita rubescens*, *Hygrophoropsis aurantiaca*, *Lepista nabularis*, *Paxillus involutus*, and *Suillus luteus* that emerged in natural habitats. In other studies, they did not influence the accumulation of Ag, Al, Ba, Ca, Cd, Co, Cr, Cu, Fe, Hg, K, Mg, Mn, Na, Ni, Sr, Pb, Rb, and Zn by *A*. *muscaria*, *P. involutus*, and *Leccinum scabrum* (Brzostowski et al. 2009, 2011a; Falandysz and Bielawski 2007; Falandysz and Brzostowski 2007; J.F., unpublished).
  Concentrations of Ni and Cr were elevated in macrofungi from serpentine soils (Aloupi et al. 2011). Interestingly, metal concentrations in fruit bodies of ectomycorrhizal fungi growing on the same site can/could be very similar over the years (Brzostowski et al. 2011b; J.B., unpublished). The influence of environmental factors was studied also by Campos et al. (2009) who, however, used an inappropriate analytical method, and the published results are not reliable—see Borovička et al. (2011). As demonstrated in a study by Cen et al. (2012), the addition of chelating agents such as ethylenediaminetetraacetic acid and nitrilotriacetate to soil in a pot study enhanced the bioaccumulation of Cd, Cu, and Pb in *Coprinus comatus*, while the addition of citric acid did not.
2. Metalliferous areas and environmental pollution: Naturally or artificially elevated levels of metals in soils usually result in greater concentrations in fruit bodies. This has been demonstrated for many elements including Au (Borovička et al. 2010a), Ag (Borovička et al. 2005, 2010b; Kojta et al. 2012), As (Larsen et al. 1998), Cd (Kalač et al. 1991; Falandysz et al. 2011), Cu (Svoboda et al. 2000), Hg (Bargagli and Baldi 1984; Falandysz et al. 2012c, 2012e), Ni (Barcan et al. 1998), Pb (Kalač et al. 1991), and Sb (Borovička et al. 2006).
3. Fungal lifestyle: Concentrations of Hg, Cd, Pb and Pb are usually greater in terrestrial saprotrophs than those in ectomycorrhizal fungi (Borovička and Řanda 2007 and many others). On the other hand, concentrations of alkali metals, Rb and Cs, are generally greater in ectomycorrhizal species (Klán et al. 1988; Řanda and Kučera 2004; see also Table 12). Some of these differences might be explained by contrasting nutrition strategy of both groups and consequently the differences in the distribution of mycelia in soil profiles (Luis et al. 2005; Christ et al. 2011).
4. Bioaccumulation of metallic elements, metalloids and non-metals (Se) in fruit bodies is highly species specific: Some macrofungi are able to accumulate elements much more effectively than others; the ability to accumulate a particular element is a characteristic feature of a particular fungal species. In various macrofungi growing above the same substrate, concentrations of elements can vary over a wide range and can differ even a thousand times from species to species (see, e.g., Lavola et al. 2011).
5. The accumulation process can be highly element specific: Macrofungi may even discriminate elements with similar properties and chemical behavior (homologues). A typical example might be hyperaccumulation/discrimination of As/Sb in *Sarcosphaera coronaria* (see below).

## Hyperaccumulation

An extraordinarily high ability of a plant or macrofungal species to accumulate a trace element in its tissues is called hyperaccumulation. To be defined as a hyperaccumulator, the metal concentration should be at least about 100 times greater than the values to be expected in nonaccumulating species on the same substrate (for discussion on this term, see Borovička et al. 2007).

*A. muscaria* was the first recognized fungal hyperaccumulator. This temperate zone species hyperaccumulates vanadium, and this also applies to its relatives such as *Amanita regalis* and *Amanita velatipes*. Whereas concentrations of V in other mushrooms hardly exceed 1 mg kg^−1^ (related to dry matter, dm), these *Amanita* spp. accumulate even hundreds of milligram per kilogram of dm. In fruit body biomass, V is present as amavadin, an eight-coordinate V complex of unclear biological function (Garner et al. 2000).

*S. coronaria*, an ectomycorrhizal ascomycete growing on calcareous bedrock in temperate climate regions, hyperaccumulates arsenic. Despite being relatively low naturally in soils, the As concentrations are commonly as high as 1,000 mg kg^−1^ dm in fruit bodies of this species (Stijve et al. 1990), with the greatest ever reported value being 7,090 mg kg^−1^ dm (Borovička 2004). The main As compound identified in biomass was methylarsonic acid (Byrne et al. 1995). Although less toxic than arsenic trioxide, it is still relatively dangerous. Therefore, the Pink Crown should not be eaten though it used to be considered edible in Europe. Hyperaccumulation of As occurs also in several Basidiomycetes (J.B., unpublished; and see also text below).

The most recently discovered hyperaccumulators are *Amanita* species of the section *Lepidella* (Borovička et al. 2007). The European *Amanita strobiliformis* like some others growing worldwide hyperaccumulates silver. Despite very low natural levels in soils (below 1 mg kg^−1^ dm), Ag concentrations in *Amanita* spp. may be more than 2,000 times greater and commonly amount to hundreds of milligram per kilogram of dry matter; the greatest reported value was 1,253 mg kg^−1^ dm. Investigation on the chemical form of Ag has revealed that *A. strobiliformis* employs cysteine-rich, low molecular weight proteins (metallothioneins) to sequester intracellular Ag in its fruit bodies and extraradical mycelium (Osobová et al. 2011).

The biological importance of elemental hyperaccumulation in macrofungi is unclear but this extraordinary feature can be thought of in theory as being attributable to the “defense hypothesis” (Boyd 2007). It has been shown in vascular plants that elemental hyperaccumulation may have several functions, including plant defense against natural enemies; at least some tests have demonstrated defense by hyperaccumulated As, Cd, Ni, Se, and Zn in plants. In view of this fact, the high Ag concentrations in hyperaccumulating macrofungal species might have some protective effect against pathogenic microfungi, bacteria, insect larvae, or gastropoda, but this is yet to be tested. Interestingly, Gryndler et al. (2012) have demonstrated that Ag-rich fungal biomass strongly influences the soil bacterial community.

## Edible mushrooms and mineral constituents

Numerous wild-grown mushrooms are more or less valued as gourmet by nations worldwide (Tüzen et al. 2007; Zhang et al. 2010; Costa-Silva et al. 2011; Li et al. 2011; Nnorom et al. 2012a; Giannaccini et al. 2012; Figs. S1–S4, Supporting material). Also, in China and elsewhere, mushrooms collected in the wild are still used in traditional medicine, e.g., *Ganoderma lucidum* (Zhao et al. 2004), which is also cultivated in China. Another example can be sclerotium (or storage tuber that is formed by dense mass of mycelium) of *Wolfiporia extensa* used in Chinese traditional medicine and of *Pleurotus tuber-regium* that are also considered a popular food (Figs. S5–S7, Supporting material). Products based on mycelium that were biotechnologically enriched in desired trace element could be considered possible nutraceuticals (food supplements), and an example can be Se-enriched mycelium of *Lenitula edodes* mushroom (Turło et al. 2010). There are growing attempts to enrich cultivated mushrooms with biologically active and medicine minerals desired in human nutrition, and typical examples are Se and Li (Zhao et al. 2004; Silva et al. 2010; Bhatia et al. 2011; Hong et al. 2011; Assunção et al. 2012).

Mushrooms are valued due to their aroma and taste, as well as their proteins, vitamins, and low fat contents, and they are considered as products rich in mineral compounds. Apart from the abundance of minerals desired in human nutrition in mushrooms, undesired potentially toxic elements including Cd and Hg also occur sometimes at elevated concentrations (Falandysz et al. 2011; Gucia et al. 2012a, b; Giannaccini et al. 2012; Jarzyńska et al. 2012a).

First of all, most of the estimated 2,000 species of edible wild mushrooms grown worldwide are poorly characterized with respect to their multi-mineral composition. Apart from the variability introduced by natural conditions (type of soil bedrock, habitat, environmental factors), little is known of the influence of the processing, storage, and cooking on the contents and changes in mineral compounds composition of mushrooms as well as their accessibility and bio-availability from mushroom meals to man (Svoboda et al. 2002; Sun et al. 2012). Certain mushrooms can be processed and cooked in various ways. An example is *Tricholoma equestre*, which is suitable for drying, pickling, salting, stewing, souring, and freezing. In a recipe on mushroom soups, “the Yellow Knights’ soup,” it is noted that the fresh specimens of *T. equestre* do not need to be blanched before soup cooking—that is the specimens, when “cleaned up, cut and well rinsed in running tap water, are poured directly into boiling bullion and boiled for 40 min”. A common practice is blanching of mushrooms (a short-time boiling) by boiling them for 2–3, 5, or 10 min and slightly salted water—depending on the cuisine recipes (Maćkiewicz and Falandysz 2012). Preservation of mushrooms by salting is a practice in China and is good for *Boletus* and other species (Figs. S8–S10 in Supporting material). Those factors can influence the content (with the possibility of leaching) and chemical species of trace elements of mushroom meals. In China, *Tricholoma matsutake* can be eaten raw, and raw *Agaricus bisporus* can be found in salads worldwide. However, this is no longer a common practice in the modern world, and eating of any mushroom raw is not recommended by the authors. When well cooked, mushrooms by volume could be important and attractive components of meals (Fig. S1 in Supporting material).

Some of the mineral constituents, including metallic elements, are macro- and micronutrients to mushrooms. The essential elements are K, P, Mg, Ca, Na, Zn, Cu, Mn, Ni, and Co, while many others have no known biological function. Selenium can be found in all mushrooms but species vary in their Se contents and a few can be considered abundant in this antioxidant (Falandysz 2008). As mentioned, example of elements that are well bioaccumulated by numerous species are Au, Ag, As, Br, Cd, Cl, Cs, Cu, Hg, Rb, Se, and Zn (BAF > 1). Lead, for which BAF is usually <1, is relatively abundant in forest topsoil in background areas and is also a common trace contaminant of mushrooms. Fungi have low potential to bioaccumulate Pb and this keeps its concentration it their flesh below actual limits of tolerable intake from foods (Chudzyński and Falandysz 2008; García et al. 2009; Frankowska et al. 2010; Jarzyńska et al. 2011, 2012b; Giannaccini et al. 2012; Jarzyńska and Falandysz 2012a, b; Malinowska et al. 2004; Rudawska and Leski 2005a, b; Szubstarska et al. 2012).

## Macro and trace mineral constituents of nutritional value

Wild-grown edible mushrooms can contain certain minerals essential to humans at relatively high concentrations, and for trace elements such as Cu and Zn, published data are available for numerous but not all species. Some other essential elements depending on species are a little less or not covered at all in literature. However, this is changing in recent years as more and high quality data become available from scientific literature. Within this mini-review, focus is given mostly to elements that are of greater nutritional/medical and toxicological significance and largely by using popular wild-grown *Boletus edulis* and *Macrolepiota procera* and cultivated *A. bisporus*, *Pleurotus ostreatus*, and some other *Pleurotus* spp. and *L. edodes* for which some analytical data were published as examples.

Metals such as K, P, Mg, Ca, Na, Cu, and Zn are among the vital ions to body liquids and tissues and are co-factors of numerous enzymes, while their concentrations in the body of healthy biota are regulated. The flesh of mushrooms can be considered as rich in K and P, and it can also be considered as abundant in trace elements such as Cu and Zn. Both Cu and Zn in the flesh of mushrooms collected from background areas are of nutritional value for humans. The situation can be different if the mushrooms emerged at sites polluted with Cu or Zn. This is because in polluted soils other toxic metals (Cd, Pb, Hg) can co-occur with Cu and Zn and can be bioaccumulated to concentrations much above typical contents of edible species and could become noxious to mushrooms (Collin-Hansen et al. 2005).

*Potassium* plays many important roles as an essential metal in vegetation. This metal occurred in caps of *B. edulis* from many sites at 25 ± 0 to 29 ± 3 g kg^−1^ dm on average, and in stipes, at 20–40 % less (Table 1). The edible cap of *M. procera* is also rich in K, and its typical concentration is 26 ± 4 to 49 ± 14 g kg^−1^ dm (Table 1), but the stipes are sometimes richer in this element (Gucia et al. 2012a, b). Wild-grown *P. tuber-regium* showed K in sclerotia (storage tuber) at a concentration one order of magnitude less compared to the fruit bodies of cultivated *Pleurotus* spp. (Table 1). Mycelium compared to fruit bodies of wild-grown mushrooms is also one order of magnitude poorer in K (Vinichuk et al. 2011). The values of concentrations in Tables 1 and 12 imply that there are similarities in K contents of fruit bodies of macromycetes, while fluctuations seem be more due to the stage of maturity of the carpophore and the site rather than to the species of the fungus (Tables 1 and 12).

**Table 1:** Potassium in mushrooms (adapted)

Species	Content (g kg^−1^ dm)			Ref.
	Mean/s ± SD	Median/s	Total range	
*B. edulis* (caps)	25 ± 0–29 ± 3	20–38	14–41	A
*M. procera* (caps)	26 ± 4–49 ± 14	25–74	17–110	B
*L. pseudoscabrum* (caps)	40 ± 3	41	34–43	C
*L. duriusculum* (caps)	37 ± 3	37	33–44	D
*L. scabrum* (caps)	34 ± 3–52 ± 4	34–52	26–59	E
*L. rufum* (caps)	35 ± 81–42 ± 45	31–41	25–50	F
*A. bisporus* (caps)	40 ± 0	–	–	G
*A. bisporus* (whole)	38 ± 1–40 ± 1	–	–	H
*A. bisporus* (canned)	0.45 ± 0.01–1.3 ± 0.1	–	–	H
*A. subrufescens* (caps)	28 ± 2–32 ± 1	–	–	I
*P. tuber-regium* (sclerotia)	3.4 ± 1.6–4.1 ± 2.4	3.2	1.3–10	J
*Pleurotus* spp. (whole)	22 ± 1–40 ± 2	–	–	K
*P. ostreatus* (caps)	31 ± 6	–	–	G
*L. edodes* (whole)	26 ± 0	–	–	K
*L. edodes* (caps)	23 ± 4	–	–	G

A (Falandysz et al. 2008a, 2011; Frankowska et al. 2010; Zhang et al. 2010); B (Kojta et al. 2011; Jarzyńska et al. 2011; Gucia et al. 2012a, b); C (Jarzyńska and Falandysz 2012a); D (Jarzyńska and Falandysz 2012b); E (Falandysz et al. 2007e; Zhang et al. 2013); F (J.F., unpublished); G (Vetter et al. 2005); H (Vetter 2003); I (Györfi et al. 2010); J (Nnorom et al. 2012b); K (Manzi et al. 1999)

*Phosphorus* is abundant in wild-grown edible mushrooms and a concentration range of 10 ± 1 to 15 ± 2 g kg^−1^ dm was reported in the caps of *M. procera*, while the concentrations (Table 2) in stipes were 40–50 % less (Gucia et al. 2012a, b). The mushroom *M. procera* is more abundant in P than certain other wild-grown species for which data are available. For example, *Leccinum duriusculum* (rare species) and *L. scabrum* showed P in caps of 5.7 and 5.7 g kg^−1^ dm (median values), and in stipes, these were 1.5 and 2.2 g kg^−1^ dm, respectively (Jarzyńska and Falandysz 2012b; Zhang et al. 2012). For *Suillus grevillei* and *Xerocomus badius*, the concentrations of P are largely similar to those of *Leccinum* spp. (Table 2), while the values for *S. grevillei* and *X. badius* in stipes were much less (Chudzyński and Falandysz 2008; Kojta et al. 2012). The concentrations reported for *Cantharellus cibarius* (whole fruit bodies) did not deviate from those reported for the species mentioned (Table 2). Also for some cultivated *Agaricus* spp., *Pleurotus* spp., and *L. edodes*, the content of P was similar to that of wild-grown species but much less in commercially processed (conserved) *A. bisporus* and also in the sclerotia of *P. tuber-regium* (Table 2).

**Table 2:** Phosphorus in mushrooms (adapted)

Species	Content (g kg^−1^ dm)			Ref.
	Mean/s ± SD	Median/s	Total range	
*M. procera* (caps)	10 ± 1–15 ± 2	10–19	7.8–24	A
*L. duriusculum* (caps)	5.8 ± 0.8	5.7	4.7–7.6	B
*L. scabrum* (caps)	6.1 ± 1.1	5.7	4.6 –8.9	C
*S. grevillei* (caps)	6.4 ± 1.3–11 ± 2	6.6–11	2.6–13	D
*X. badius* (caps)	3.9 ± 1.4–6.4 ± 1.1	3.6–6.0	2.1–8.5	E
*C. cibarius* (whole)	3.0 ± 0.2–4.8 ± 0.7	3.0–4.8	2.6–6.0	F
*A. bisporus* (caps)	12 ± 2	–	–	G
*A. bisporus* (whole)	10 ± 0–10 ± 0	–	–	H
*A. bisporus* (conserved)	3.8 ± 0.1–4.5 ± 0.1	–	–	H
*A. subrufescens* (caps)	10 ± 1–13 ± 0	–	–	I
*P. ostreatus* (caps)	7.0 ± 3.0	–	–	G
*P. tuber-regium* (sclerotia)	1.9 ± 0.9–2.5 ± 1.1	2.2	0.56–3.9	I
*L. edodes* (caps)	7.4 ± 1.6	–	–	G

A (Jarzyńska et al. 2011; Kojta et al. 2011; Gucia et al. 2012a, b); B (Jarzyńska and Falandysz 2012b); C (Zhang et al. 2013); D (Chudzyński and Falandysz 2008; Falandysz et al. 2012a); E (Kojta et al. 2012); F (Falandysz et al. 2012b); G (Vetter et al. 2005); H (Vetter 2003); I (Györfi et al. 2010); J (Nnorom et al. 2012b)

*Sodium* in the form of NaCl is added to many food products and meals, and the problem usually is its excessive intake but it is usually not in deficit in the human body. Mushrooms as organic food can be considered low in this element. For example, in the caps of *B. edulis*, the median sodium concentrations of 100 to 380 mg kg^−1^ dm and in *M. procera* at 40 to 500 mg kg^−1^ dm have been reported (Table 3). Stipes of *B. edulis* and *M. procera* are about twice more abundant in Na than the caps (Falandysz et al. 2011; Gucia et al. 2012a, b). The values of Na for *B. edulis* and *M. procera* seem to agree with the concentrations reported in some cultivated mushrooms, i.e., three *Pleurotus* spp. and *L. edodes* and of some other species but preserved (in brine in tin) *A. bisporus* is rich of hidden Na (Tables 3 and 12).

**Table 3:** Sodium in mushrooms (adapted)

Species	Content (mg kg^−1^ dm)			Ref.
	Mean/s ± SD	Median/s	Total range	
*B. edulis* (caps)	150 ± 140–360 ± 70	100–380	17–550	A
*M. procera* (caps)	44 ± 14–410 ± 300	20–500	15–830	B
*L. pseudoscabrum* (caps)	520 ± 240	560	170–860	C
*L. duriusculum* (caps)	340 ± 130	310	190–660	D
*L. scabrum* (caps)	270 ± 87–530 ± 240	270–480	120–1,300	E
*L. rufum* (caps)	60 ± 41–620 ± 260	30–550	23–1,200	F
*A. bisporus* (caps)	700 ± 120	–	–	G
*A. bisporus* (whole)	860 ± 23–850 ± 25	–	–	H
*A. bisporus* (conserved)	16,000 ± 1,200–25,000 ± 0	–	–	H
*A. subrufescens* (caps)	110 ± 5–220 ± 40	–	–	J
*Pleurotus* spp. (whole)	220 ± 60–1,400 ± 460	–	–	G
*P. ostreatus* (caps)	270 ± 41	–	–	G
*L. edodes* (whole)	1,000 ± 10	–	–	J
*L. edodes* (caps)	440 ± 91	–	–	G

A (Falandysz et al. 2008a, 2011; Frankowska et al. 2010; Zhang et al. 2010); B (Jarzyńska et al. 2011; Kojta et al. 2011; Gucia et al. 2012a, b); C (Jarzyńska and Falandysz 2012a); D (Jarzyńska and Falandysz 2012b); E (Falandysz et al. 2007e; Zhang et al. 2013); F (J.F., unpublished); G (Vetter et al. 2005); H (Vetter 2003); I (Györfi et al. 2010); J (Manzi et al. 1999)

*Magnesium* occurs in a very similar concentration in many mushrooms both wild grown and cultivated (Table 4). Caps of *B. edulis* contained Mg at 680 to 930 mg kg^−1^ dm and less than half this concentration were in stipes (Falandysz et al. 2011). Magnesium contents of processed *A. bisporus* is about half the contents of unprocessed mushrooms, and it seems that large amounts of elements can be lost from this species when boiled in saline water (Table 4).

**Table 4:** Magnesium in mushrooms (adapted)

Species	Content (mg kg^−1^ dm)			Ref.
	Mean/s ± SD	Median/s	Total range	
*B. edulis* (caps)	590 ± 80–960 ± 94	680–930	330–1,200	A
*M. procera* (caps)	860 ± 900–2,000 ± 200	840–2,000	750–2,400	B
*L. pseudoscabrum* (caps)	1,200 ± 56	1,200	1,000–1,200	C
*L. duriusculum* (caps)	1,100 ± 82	1,100	920–1,200	D
*L. scabrum* (caps)	990 ± 740–1,200 ± 100	1,000–1,200	650–1,300	E
*L. rufum* (caps)	900 ± 600–1,300 ± 1,300	820–1,200	600–1,500	F
*A. bisporus* (caps)	1,400 ± 990	–	–	G
*A. bisporus* (whole)	1,100 ± 25–1,100 ± 38	–	–	H
*A. bisporus* (conserved)	390 ± 20–470 ± 22	–	–	H
*A. subrufescens* (caps)	1,000 ± 95–1,300 ± 23	–	–	I
*Pleurotus* spp. (whole)	1,300 ± 70–2,000 ± 14	–	–	J
*P. ostreatus* (caps)	1,400 ± 320	–	–	G
*L. edodes* (whole)	1,200 ± 52	–	–	J
*L. edodes* (caps)	1,300 ± 180	–	–	G

A (Falandysz et al. 2008a, 2011; Frankowska et al. 2010; Zhang et al. 2010); B (Jarzyńska et al. 2011; Kojta et al. 2011; Gucia et al. 2012a, b); C (Jarzyńska and Falandysz 2012a); D (Jarzyńska and Falandysz 2012b); E (Falandysz et al. 2007e; Zhang et al. 2013); F (J.F., unpublished); G (Vetter et al. 2005); H (Vetter 2003); I (Györfi et al. 2010); J (Manzi et al. 1999)

*Calcium* in caps of *B. edulis*, *M. procera*, and *Leccinum* spp. are in similar concentrations and more Ca have been reported in cultivated mushrooms (Table 5). An exception can be preserved (in brine in tin) *A*. *bisporus* that contained Ca at concentrations >2,000 mg kg^−1^ dm, but this could be due to Ca from industrial brine (Table 5). In *B. edulis*, *M. procera*, and *Leccinum* spp., both caps and stipes are similarly rich in Ca (Falandysz et al. 2011; Gucia et al. 2012a, b; Jarzyńska and Falandysz 2012a, b; Zhang et al. 2012).

**Table 5:** Calcium in mushrooms (adapted)

Species	Content (mg kg^−1^ dm)			Ref.
	Mean/s ± SD	Median/s	Total range	
*B. edulis* (caps)	38 ± 25–190 ± 110	29–170	5.6–420	A
*M. procera* (caps)	68 ± 81–320 ± 280	54–290	18–970	B
*L. pseudoscabrum* (caps)	110 ± 36	110	67–220	C
*L. duriusculum* (caps)	88 ± 26	87	59–150	D
*L. scabrum* (caps)	54 ± 20–140 ± 49	52–150	27–200	E
*L. rufum* (caps)	60 ± 30–170 ± 50	50–170	20–400	F
*A. bisporus* (caps)	1,400 ± 530	–	–	G
*A. bisporus* (whole)	860 ± 72–890 ± 0	–	–	G
*A. bisporus* (conserved)	2,100 ± 140–2,600 ± 33	–	–	G
*A. subrufescens* (caps)	570 ± 39–1,100 ± 57	–	–	H
*Pleurotus* spp. (whole)	190 ± 21–490 ± 15	–	–	I
*P. ostreatus* (caps)	820 ± 110	–	–	G
*L. edodes* (whole)	420 ± 20	–	–	I
*L. edodes* (caps)	1,100 ± 130	–	–	G

A (Falandysz et al. 2008a, 2011; Frankowska et al. 2010; Zhang et al. 2010); B (Jarzyńska et al. 2011; Kojta et al. 2011; Gucia et al. 2012a, b); C (Jarzyńska and Falandysz 2012a); D (Jarzyńska and Falandysz 2012b); E (Falandysz et al. 2007e; Zhang et al. 2013); F (J.F., unpublished); G (Vetter 2003); H (Györfi et al. 2010); I (Manzi et al. 1999)

*Copper* in caps of *B. edulis* from many sites was at 24 to 79 mg kg^−1^ dm (median) (Table 6), and the stipes contained less than half of these amounts (Falandysz et al. 2011). Some quality data on Cu for *B. edulis* are also available from other sources (Collin-Hansen et al. 2002; Řanda and Kučera 2004). The species *Boletus tomentipes* collected in Yunnan (China) seems to be less rich in Cu compared to *B. edulis* (Table 6). Some mushrooms of the genus *Leccinum*, which are related to the genus *Boletus*, showed Cu in a similar concentration, and *Leccinum rufum* with Cu contents of 45 to 97 (median) mg kg^−1^ dm in caps (Table 6) seems to be an exception. The stipes of *Leccinum* mushrooms contained around half Cu compared to the caps (Jarzyńska and Falandysz 2012a, b; J.F., unpublished; Zhang et al. 2012).

**Table 6:** Copper in mushrooms (adapted)

Species	Content (mg kg^−1^ dm)			Ref.
	Mean/s ± SD	Median/s	Total range	
*B. edulis* (caps)	26 ± 11–57 ± 24	24–79	6.9–94	A
*B. edulis* (whole)	28 ± 14–64 ± 11	35	11–77	B
*B. tomentipes* (whole)	14 ± 1	–	11–14	C
*L. pseudoscabrum* (caps)	29 ± 9	27	19–48	D
*L. duriusculum* (caps)	18 ± 6	16	8.4–34	E
*L. scabrum* (caps)	21 ± 8–30 ± 16	20–29	9.1–63	F
*L. rufum* (caps)	73 ± 22–100 ± 27	45–95	22–150	G
*M. procera* (caps)	110 ± 200–200 ± 60	83–230	52–360	H
*M. procera* (whole)	120 ± 23–210 ± 29	170	98–240	B
*A. bisporus* (caps)	40 ± 10	–	–	I
*A. bisporus* (whole)	58 ± 2–65 ± 1	–	–	J
*A. bisporus* (conserved)	15 ± 0–15 ± 1	–	–	J
*A. subrufescens* (caps)	63 ± 3–220 ± 4	–	–	K
*P. ostreatus* (caps)	19 ± 9	–	–	I
*L. edodes* (caps)	13 ± 2	–	–	I

A (Falandysz et al. 2008a, 2011; Frankowska et al. 2010; Zhang et al. 2010); B (Giannaccini et al. 2012); C (Li et al. 2011); D (Jarzyńska and Falandysz 2012a); E (Jarzyńska and Falandysz 2012b); F (Falandysz et al. 2007e; Zhang et al. 2013); G (J.F., unpublished); H (Falandysz et al. 2008b; Jarzyńska et al. 2011; Kojta et al. 2011; Gucia et al. 2012a, b); I (Vetter et al. 2005); J (Vetter 2003); K (Györfi et al. 2010)

In the caps of *M. procera*, the contents of Cu (many individual samples from background sites) were between 83 and 230 (median) mg kg^−1^ dm (Table 6). The Italian authors examined caps and inedible stipes together (whole fruit bodies), and the Cu contents were similar as in the caps above, i.e., at 170 (median) mg kg^−1^ dm (Table 6). In stipes of *M. procera*, the contents of Cu can be the same as in caps or lower (Gucia et al. 2012a). Copper is an essential element and its uptake to some degree can be regulated by the fungus. Data reviewed above on Cu in *B. edulis* and *M. procera* are based on large sets of results and mushrooms collected from several sites. The data showed that apart from being statistically significant (0.05 < *p* < 0.01), spatial variations were noted for a particular species, while the contents of Cu can vary between species too. Hence, the significance of a particular species of mushrooms as a potential (and local) source of Cu to man can vary between regions, while differences in Cu content between species occur also.

*Zinc* in *B. edulis* was reported at between 100 and 210 mg kg^−1^ dm (median for caps and whole fruit bodies) and much lower values in *B. tomentipes* (Table 7). The content of Zn in stipes of *B. edulis* is usually about half or even less than in caps (Falandysz et al. 2008a). Similarly, the stipe of *M. procera* on average contained less Zn, about half the content in the cap (Gucia et al. 2012a). Cultivated *A. bisporus*, *P. ostreatus*, and *L. edodes* showed less Zn than *A. subrufescens* and in other wild-grown species reviewed in this paper (Table 7), but the number of data available for cultivated mushrooms is less. The edible wild-grown and cultivated mushrooms can be considered as foods relatively abundant in/with K, Cu, and Zn, and taking into consideration that these elements are also abundant in many staple foods, clearly there is no need to bio-fortify any mushroom with them.

**Table 7:** Zinc in mushrooms (adapted)

Species	Content (mg kg^−1^ dm)			Ref.
	Mean/s ± SD	Median/s	Total range	
*B. edulis* (caps)	120 ± 27–210 ± 71	110–210	63–360	A
*B. edulis* (whole)	74 ± 6–140 ± 20	100	43–350	B
*B. tomentipes* (whole)	20 ± 1	–	19–23	C
*L. pseudoscabrum* (caps)	240 ± 96	210	93–420	D
*L. duriusculum* (caps)	150 ± 31	150	95–200	E
*L. scabrum* (caps)	110 ± 24–240 ± 54	220–250	130–370	F
*L. rufum* (caps)	91 ± 35–240 ± 91	82–240	41–470	G
*M. procera* (caps)	74 ± 22–190 ± 45	73–200	55–240	H
*M. procera* (whole)	71 ± 9–200 ± 8	85	61–280	B
*A. bisporus* (caps)	65 ± 10	–	–	I
*A. bisporus* (whole)	60 ± 0–62 ± 0	–	–	J
*A. bisporus* (conserved)	60 ± 1–86 ± 4	–	–	J
*A. subrufescens* (caps)	200 ± 18–320 ± 11	–	–	K
*P. ostreatus* (caps)	77 ± 3	–	–	I
*L. edodes* (caps)	88 ± 11	–	–	I

A (Falandysz et al. 2008a, 2011; Frankowska et al. 2010; Zhang et al. 2010); B (Giannaccini et al. 2012); C (Li et al. 2011); D (Jarzyńska and Falandysz 2012a); E (Jarzyńska and Falandysz 2012b); F (Falandysz et al. 2007e; Zhang et al. 2013); G (J.F., unpublished); H (Falandysz et al. 2008b; Jarzyńska et al. 2011; Kojta et al. 2011; Gucia et al. 2012a, b); I (Vetter et al. 2005); J (Vetter 2003); K (Györfi et al. 2010)

## Selenium and lithium in mushrooms as examples of highly/specifically desired micronutrients

*Selenium* is frequently deficient in soils and foods worldwide, and in wild-grown mushrooms, its content varies depending on the species (Falandysz 2008; Rayman 2008; Jarzyńska and Falandysz 2011a, b). This element is highly potent and has great health impacts. It is needed in the synthesis of mammalian selenoenzymes, and glutathione peroxidase-3 and selenoprotein-P are dominant Se compounds secreted in the blood. There are many factors for which Se plays a role, and these need to be considered when deciding on the optimal human supplementation with dietary Se (or its species) including a key co-occurrence in vitamin E and other antioxidants but also other mineral constituents such as hazardous As, Cd, and Hg (Jarzyńska and Falandysz 2011a, b).

A good bio-available source of Se from foods is needed for the full expression of selenoproteins with antioxidant function and particularly in diseases associated with oxidative stress. Dietary daily intake of Se for humans is recommended at 57 μg (range 30–85 μg), but this sometimes can be insufficient for the expression of selenoprotein-P, and its high adequate intake rate is estimated for approximately 100–200 μg per day (Rayman 2008). The margin of safety for Se is narrow. In the past, overdosing causing selenosis happened to horses (Se poisoning) grazing on Se-hyperaccumulating plants and humans eating foods from areas contaminated with Se-containing waste.

Of around 200 edible species reviewed (belonging to 21 families and 56 genera), most were relatively low in Se, i.e., *<*1 mg kg^−1^ dm (Falandysz 2008). The fruit bodies of some species of wild-grown edible mushrooms are naturally rich in selenium. *Albatrellus pes-caprae* with ~200 mg kg^−1^ dm on the average (maximum up to 370 mg kg^−1^ dm) is the richest among the species surveyed. Several other representatives of the genus *Albatrellus* are also abundant in Se. Of the most popular edible wild-grown mushrooms, the *B. edulis* is considered abundant in Se as well; on average, it contains ~20 mg kg^−1^ dm (maximum of up to 70 mg kg^−1^ dm). Some species of the genus *Boletus*, such as *Boletus pinophilus*, *Boletus aereus*, *Boletus reticulatus*, and *Boletus erythropus* can also accumulate considerable amounts of selenium. Some other species relatively rich in Se include *A. strobiliformis*, which contains, on average, ~20 mg kg^−1^ dm (up to 37 mg kg^−1^ dm), and the *Lycoperdon* spp., with an average of ~5 mg kg^−1^ dm (Falandysz 2008). The mushroom *M. procera* with a range of ~3–5 to *<*10 mg Se kg^−1^ dm in caps can be also be considered to be rich in this element (Tüzen et al. 2007; Falandysz 2008; Giannaccini et al. 2012).

For several wild-grown species of the genus *Agaricus*, the selenium content (~5 mg kg^−1^ dm) is much greater than that from cultivated Champignon mushroom; these include *Agaricus bitorquis*, *Agaricus campestris*, *Agaricus augustus*, *Agaricus macrosporus*, and *Agaricus sylvaticus* (Falandysz 2008). In cultivated commercially available *A. bisporus*, the contents of Se were between 1.9 ± 0.1 and 3.7 ± 0.9 mg kg^−1^ dm (Vetter 2003) and 0.28 ± 0.68 and 1.4 ± 0.1 mg kg^−1^ dm (Costa-Silva et al. 2011). Canned (preserved) commercially available *A. bisporus* was much less in Se (<0.5 mg kg^−1^ dm) compared to fresh mushrooms examined by Vetter (2003). And in recent studies of cultivated *P. ostreatus* from the European market, Se was at 0.10 ± 0.01 to 0.26 ± 0.20 mg kg^−1^ dm (Costa-Silva et al. 2011), while the Se content of samples from the USA was 0.2 mg kg^−1^ dm (Hong et al. 2011).

Different Se compounds can be utilized in the formation of Se enzymes, and the most desired is Se cysteine, which is low in plant or mushroom proteins compared to Se methionine. In mushrooms, several Se compounds have been identified including selenomethionine, selenocysteine, Se methylselenocysteine, selenite, seleno-polysaccharides, and several other unidentified seleno-compounds that occur in varying proportions (Falandysz 2008).

In early attempts to produce Se-enriched edible mushrooms (using *A. bisporus* and *L. edodes*), inorganic Se salts were used as additives to the substratum (Falandysz 2008). Compost fortified with inorganic Se (Na_2_SeO_3_) at a rate of 8.1 and 14.4 mg kg has been used, and the Se-enriched *Pleurotus eryngii* contained, respectively, 4.6 and 9.3 mg Se kg^−1^ dm (<1.5 mg kg^−1^ dm in reference fruit bodies) (Estrada et al. 2009).

Recently, a new approach to obtain Se-enriched foods is by the use of Se-laden vegetation instead of inorganic Se in the cultivation of mushrooms. In a study by Hong et al. (2011), the fruit bodies of *P. ostreatus* emerged in Se-laden plant compost contained 2.9 mg Se kg^−1^ dm. In experiments with *Pleurotus florida* cultivated using Se-rich wheat straw (Se in straw was at 26 ± 0, and grains contained 89 ± 1 mg kg^−1^ dm) from the seleniferous belt of Punjab of India, the Se content of fruit bodies was spectacularly high at 130 ± 2 mg kg^−1^ dm, and in reference mushrooms, it was 0.16 ± 0.01 mg kg^−1^ dm (Bhatia et al. 2011).

Also available are data on the impact of boiling/blanching on the Se content of *A. bisporus* (44 % loss) and in *Lactarius torminosus* (32 % loss). Data on Se bioavailability from a meal containing *B. edulis* (weak bioavailability) and cultivated *A. bisporus* and wild-grown *B. edulis* (weak bioavailability) have been reported too. The intake of Se-enriched *A. bisporus* (53 mg kg^−1^ dm) increased the activities of the liver and mammary glutathione *S*-transferase (GCT) (cited after Falandysz 2008).

In a recent study on the availability of Se to rats that were fed with Se-enriched *P. ostreatus* (feed contained 0.15, 0.30, or 0.45 mg Se kg^−1^) and ordinary feed fortified with sodium selenate (0.15 Se kg^−1^), the profile of some Se-containing proteins in plasma was similar for all animals, while the rate of Se absorption was not given. Selenium from mushroom was the highest in a peptide of 8 kDa size, while the feed with mushroom component resulted in a greatest content of this peptide in rats’ blood (7 μg Se L^−1^) compared to 2–5 μg Se L^−1^ from other diets (Silva et al. 2010).

A mushroom meal that contains 0.005–0.05 mg Se kg^−1^ wet weight (many species) when consumed in quantities of 100 or 300 g—assuming a limited availability of Se from mushrooms (circa 15–25 %) and its loss (circa 40 %) during mushroom blanching—can provide, respectively, circa 0.6–6 and 1.8–18 μg of bioavailable Se, respectively. Hence, if the values of the recommended daily intake of 57 μg Se (range 30–85 μg) and of high adequate intake of 100–200 μg are taken into account, the species of wild-grown mushrooms rich in Se and also cultivated mushrooms that are well bio-enriched in Se can be considered as good sources of this element for mushroom fanciers. From *B. edulis* and *B. reticulatus* with an average content of 20 mg Se kg^−1^ dm, the intake of bioaccessible Se is 24 μg (for a 100-g mushroom portion) and 72 μg (for a 300-g portion), and intake from *B. pinophilus* can be twofold greater. And if *Boletus* spp. is prepared in a manner allowing only on a negligible if any loss of “body liquid”—and so also of minerals—out of a meal (e.g., a specific Chinese or other cuisine recipes), certainly the intake will be higher. The problem in assessing intake is the fact that the same mushroom species when processed or cooked in various ways, the leakages/leaching of minerals could be anticipated. Also, the contents of minerals in industrially processed mushrooms can vary significantly from what is in fresh mushrooms, and a typical example is the data for fresh and preserved *A. bisporus* (Vetter 2003).

*Lithium* is a trace mineral in biota, and the lithium salt, Li_2_CO_3_, is therapeutic for neurodisorders (i.e., as a psychiatric medicine) for many years, but a deeper insight of its role has not been discovered. Mushrooms seem to be low in this element. In a study by Vetter (2005), the Li content of 14 taxa of mushrooms was between 0.064 ± 0.039 and 0.19 ± 0.13 mg kg^−1^ dm and higher amounts were found in *A. strobiliformis* (0.52 ± 0.55 mg kg^−1^ dm), *Craterellus cornucopioides* (0.116 ± 0.70 mg kg^−1^ dm), and *Psathyrella candolleana* (0.39 ± 0.38 mg kg^−1^ dm). However, entirely different data—with a range from 0.030 to 12 mg kg^−1^ dm (for some species, only a single result reported as single specimen was examined)—have been reported recently for *Russula virescens*, *Trogia* spp., *Lactarius hygrophoroides*, *Thelephora vialis*, *Russula lepida*, *Amanita exitialis*, and *T. matsutake* collected in the northern parts of Yunnan in China (Yin et al. 2012). In that study, except for *Trogia* spp*.*, all other species contained Li at >1.0 and most at >2.0 mg kg^−1^ dm, and this needs to be clarified in further studies.

In a study by Giannaccini et al. (2012), the Li content of *B. edulis* was at 0.2 ± 0.2 (median 0.1 and range 0.04–0.9) mg kg^−1^ dm and of *M. procera* at 0.3 ± 0.4 (median 0.1 and range 0.03–1.6) mg kg^−1^ dm, and these results agree with the findings for other mushrooms cited above as reported by Vetter (2005). In the only study on bio-enrichment of mushrooms with Li known, the species *P. ostreatus* cultivated in the substratum (coffee husk; 94 mg Li kg^−1^ dm) fortified with LiCl (62.5 to 500 mg kg^−1^) accumulated this element in fruit bodies and as the authors claim: “Li concentration in mushrooms was directly influenced by increasing LiCl concentration in the substrate (*p* < 0.05)” (Assunção et al. 2012). However, the Li content of nonenriched *P. ostreatus* in that study was around 50 mg kg^−1^ dm (no clear figures were given), and this is about the highest Li concentration reported for mushrooms so far. One thing noticed, however, is that the accessibility (in vitro gastrointestinal digestion) of Li was higher from bio-enriched mushrooms than from common (nonenriched) mushrooms and, in practice, is not acceptable for use in psychiatric drug (Li_2_CO_3_) (Assunção et al. 2012).

## Toxic mineral constituents in mushrooms (Hg, As, Cd, Pb, Ag)

Among the hazardous minerals that occur naturally in substrates decomposed by fungi and in the flesh of mushrooms are Hg, As, Cd, Pb, and Ag. Metallic elements such as Hg, Cd, Pb, and Ag and the metalloid As, and other, due to mining, metallurgy, use, wastes, and recovery processes, are classified as environmental pollutants. Elevated contents of Hg, Cd, Pb, and Ag in mushrooms were confirmed at polluted sites (see below).

*Mercury* can be efficiently bioaccumulated by many mushrooms and especially when it is present in small concentrations in forest soils, e.g., for caps of *M. procera,* the BAF values vary from 16 ± 6 to 220 ± 110 (total range from 0.52 to 470), and for *B. edulis*, these were from 41 ± 6 to 130 ± 39 (total variation between 13 and 170) (Falandysz et al. 2007a; Falandysz and Gucia 2008). The problem of Hg in mushrooms has been evaluated in detail for many species from different countries in recent times (Falandysz and Chwir 1997; Falandysz 2002; Falandysz and Bielawski 2001, 2007; Alonso et al. 2000; Falandysz et al. 2001a, 2002a, b, c, d, 2003a, b, c, d, 2004, 2007a, b, f, 2012c, d, e, f, 2013; Tüzen and Soylak 2005; Cocchi et al. 2006; Falandysz and Brzostowski 2007; Chudzyński et al. 2009, 2011; Melgar et al. 2009; Nasr and Arp 2011; Pilz et al. 2011; Reider et al. 2011; Chojnacka et al. 2012; Drewnowska et al. 2012a, b; Giannaccini et al. 2012; Maćkiewicz and Falandysz 2012; Nnorom et al. 2012a).

Data on the rates of highly neurotoxic methylmercury (MeHg) bioaccumulation by mushrooms vary between studies, and some have shown that it is a form more bioavailable compared to inorganic forms (considered as “total” Hg). MeHg is less abundant than the inorganic Hg, both in the wild-growing and cultivated mushrooms, i.e., it is found between ~2 and 60 % of total Hg, though the proportions vary depending on the investigator, concentration, and analytical method (Table 8) (Falandysz 2010; Fischer et al. 1995; Pilz et al. 2011; Reider et al. 2011).

**Table 8:** Mercury in mushrooms (adapted)

Species	Content (mg kg^−1^ dm)			Ref.
	Mean/s ± SD	Median/s	Total range	
*B. edulis* (caps)	1.2 ± 1.4–7.6 ± 3.1	0.73–6.3	0.02–14	A
*B. edulis* (whole)	2.7	–	1.0–4.3^a^	B
*B. edulis* (whole)	1.9 ± 1.0–4.5 ± 1.0	1.9–4.6	1.0–6.1	C
*B. edulis* (h/rfb)	3.3 ± 2.4/2.0 ± 1.2	–	–	D
*M. procera* (caps)	1.1 ± 1.0–8.4 ± 7.4	1.3–7.4	0.05–22	E
*M. procera* (whole)	2.4	–	1.5–3.3^a^	B
*M. procera* (whole)	1.4 ± 0.3–4.0 ± 0.2	1.6–4.0	1.4–4.2	C
*M. procera* (h/rfb)	2.6 ± 1.2/1.6 ± 0.7	–	–	D
*L. pseudoscabrum* (caps)	0.34 ± 0.17	0.33	0.12–0.65	F
*L. scabrum* (caps)	0.38 ± 0.23–1.2 ± 0.4	0.36–1.2	0.072–2.0	H
*L. scabrum* (h/rfb)	0.57 ± 0.23/0.44 ± 0.31	–	–	D
*L. rufum* (caps)	0.27 ± 0.07–1.3 ± 0.6	0.28–1.3	0.16–2.2	I

A (Falandysz et al. 2007a, 2011; Frankowska et al. 2010; Zhang et al. 2010); B (Cocchi et al. 2006); C (Giannaccini et al. 2012); D (Melgar et al. 2009); E (Falandysz et al. 2007b; Falandysz and Gucia 2008); F (Jarzyńska and Falandysz 2012a); G (Jarzyńska and Falandysz 2012b); H (Falandysz et al. 2007e); I (Falandysz et al. 2012d)

*h* hynenophore, *rfb* rest of fruit body

^a^95 % confidence interval

Mushrooms *B. edulis* and *M. procera* even when they emerged at background sites in Italy, Poland, and Spain can contain total Hg at elevated concentrations, i.e., for caps, was at 1.2 ± 1.4 to 7.6 ± 3.1 (total range 0.02 to 14) mg kg^−1^ dm and, for whole fruit bodies, at 1.2 ± 0.7 to 8.4 ± 7.4 (0.05 to 22) mg kg^−1^ dm, respectively (Falandysz et al. 2007a, b; Melgar et al. 2009; Giannaccini et al. 2012). They can be a source of elevated Hg intake. For example, in a study of *M. procera*, the median Hg concentration in caps varied for the sites between 1.3 and 7.0 mg kg^−1^ dm (0.13 and 0.7 mg kg^−1^ fresh weight; assuming water content is 90 %). The estimated intake of Hg resulting from the consumption of 300 or 500 g portions of caps was assessed as 39–210 and 65–350 μg, and this is equivalent to 0.65–3.5 and 1.1–5.8 μg kg^−1^ body mass (an adult of 60 kg bm) (Gucia et al. 2012b). It was concluded, that if taken into account, that provisionally tolerable weekly intake (PTWI) of Hg is 300 μg (equivalent to 5 or 0.7 μg kg^−1^ bm per day), and a reference dose of 0.3 μg kg^−1^ bm per day, Hg in caps of *M. procera* for some pristine areas might be of concern especially if eaten by fanciers frequently in the mushrooming season.

Both *B. edulis* and of *M. procera* apart from Hg can also contain Se (especially *B. edulis*) at high concentrations (see above), and this can have protective roles against Hg in biological activity but no facts are known. The accessibility and bioavailability of Hg from mushroom meals is unknown.

Wood-decaying mushroom species are low in Hg compared to the numerous soil mushrooms, and good examples are *Pleurotus* spp. with *P. ostreatus* containing between 0.028 ± 0.008 and 0.031 ± 0.011 in caps and 0.028 ± 0.006 and 0.0370 ± 0.005 mg kg^−1^ dm in stipes (Nnorom et al. 2012a). The sclerotia of wood-decaying *P. tuber-regium* are low in Hg and did contain from 0.024 ± 0.036 to 0.048 ± 0.030 mg kg^−1^ dm (Nnorom et al. 2012a). Also wood-decaying species such as *A. solidipes* that has of root-like rhizomorphs (tentacles, bootlaces) can be more abundant in Hg—specimens collected from two background areas of Poland showed Hg in caps of 0.020 ± 0.008 to 0.070 ± 0.020 mg kg^−1^ dm, on average, and significantly more, i.e., between 0.11 ± 0.03 and 0.30 ± 0.07 mg kg^−1^ dm was observed for specimens from ten other background sites (Falandysz et al. 2012e).

*Arsenic* in a real environment (nature) is the most hazardous inorganic element due to the carcinogenic risk, and there are no safe levels of arsenic. Different mushrooms can respond in different ways for the same chemical elements contained in the substratum where they live and a good example seems to be just a case of As. Many mushrooms are usually low in As (well <0.5 mg kg^−1^ dm) (Stijve et al. 1990; Giannaccini et al. 2012; Li et al. 2011; Liu et al. 2012). Some mushrooms are specifically rich in this metalloid, i.e., *Laccaria amethystina* with up to 250 mg kg^−1^ dm and *Laccaria fraterna* with up to 270 mg kg^−1^ dm (Stijve et al. 1990). Arsenic in *S. coronaria* can be at concentrations of up to 2,000 mg kg^−1^ dm and is stored mainly in the form of methylarsonic acid (Byrne et al. 1995). Arsenic accumulated by *L. amethystina* in the flesh was in the form of dimethylarsinic acid (the most identified form), methylarsonic acid, trimethylarsine oxide, arsenic acid, and arsenobetaine and insoluble in water and unidentified arsenicals contributed to total As in 15 to 32 % (Larsen et al. 1998; Stijve et al. 1990). At a site contaminated with As in Denmark, the metalloid content of *L. amethystina* was up to 1,400 mg kg^−1^ dm and dimethylarsinic acid dominated (at 68–74 % of total As) and the As content of *L. amethystina* at a reference site was between 23 and 77 mg kg^−1^ dm (Larsen et al. 1998).

Mushrooms that bioaccumulate As include *Sarcodon imbricatus*, *Entoloma lividum*, *Lycoperdon perlatum*, and some *Agaricus* spp. (Byrne et al. 1995). Arsenobetaine that is the major As compound in *S. imbricatus*, *A*. *sylvaticus*, and *A. moelleri* is considered much less toxic than many other naturally occurring types of arsenic. In *E. lividum*, the major arsenicals are the arsenite and the arsenate (Byrne et al. 1995). At the gold mine site contaminated with As in Yellowknife of Canada, the metalloid content of non-As accumulators was elevated, i.e., at 8.3 mg kg^−1^ dm in *L. scabrum*, 14 mg kg^−1^ dm in *Psathyrella candolleana*, and 36 mg kg^−1^ dm in *P. involutus* (Koch et al. 2000). In two other species, potential As accumulators, the concentrations were 410 mg kg^−1^ dm in *C. comatus* and 1,000 mg kg^−1^ dm in *Lycoperdon pyriforme.* In certain mushrooms, arsenobetaine was dominant as water-soluble arsenic compound, while in other species several compounds have been found and the proportions of arsenicals contained varied between them (Koch et al. 2000).

Cultivated *A. bisporus* when they emerge at the substratum fortified with as much as 1,000 mg As kg^−1^ dm (added as As_2_O_5_) weakly accumulated this metalloid, i.e., the value of BAF was 0.0009. In a control experiment, *A. bisporus* cultivated in a typical (clean) substratum that contained 3.8 mg As kg^−1^ dm [several As(III) and As(V) compounds were determined] contained in the flesh 0.50 mg As kg^−1^ dm, and BAF for total As was 0.13 (Soeroes et al. 2005). Cultivated mushrooms such as *A. bisporus*, *Agaricus* spp*.*, *P. ostreatus*, *P. florida*, *P. eryngii*, *P. ostreatus*, *Pleurotus salmoneo-stramineus,* and *L. edodes* purchased in Brazil contained As in concentrations between 0.009 and 0.210 mg kg^−1^ dm (Maihara et al. 2008).

*Cadmium* as indicated earlier is well bioaccumulated and can be found at elevated concentrations in wild-grown mushrooms. This metal in the flesh of edible wild mushrooms is among the toxic substances of concern. The maximum concentration of Cd permitted by the European Union regulation in cultivated *A. bisporus*, *P. ostreatus*, and *L. edodes* is 0.20 mg kg^−1^ fw (equivalent to 2.0 mg kg^−1^ dm, assuming 90 % moisture), and for other mushrooms, it is 1.0 mg kg^−1^ fw (10 mg kg^−1^ dm) (cited after Gucia et al. 2012b).

Wild-grown mushrooms frequently contain Cd in a concentration exceeding 2.0 mg kg^−1^ dm but rarely above 10 mg kg^−1^ dm. An example is the highly valued and tasty *B. edulis* and *M. procera* mushrooms for which several data are available and for numerous and also for some species of the genus *Leccinum* (Table 9). Cadmium concentration exceeding 10 mg kg^−1^ dm can be observed for a few individual samples at unpolluted areas, but at areas contaminated with Cd, this threshold can be exceeded frequently.

**Table 9:** Cadmium in mushrooms (adapted)

Species	Content (mg kg^−1^ dm)			Ref.
	Mean/s ± SD	Median/s	Total range	
*B. edulis* (caps)	3.8 ± 2.8–18 ± 15	2.6–11	0.23–52	A
*B. edulis* (whole)	4.0	–	3.0–4.9^a^	B
*B. edulis* (whole)	2.0 ± 1.6–3.4 ± 1.8	1.6–3.4	0.5–5.3	C
*M. procera* (caps)	0.63 ± 0.20–4.9 ± 3.0	0.49–3.7	0.28–11	D
*M. procera* (whole)	1.3	–	0.77–1.0^a^	B
*M. procera* (whole)	0.7 ± 0.3–7.6 ± 0.5	0.7–7.8	0.3–7.9	C
*L. pseudoscabrum* (caps)	3.3 ± 2.1	2.9	1.1–8.5	D
*L. duriusculum* (caps)	1.5 ± 0.5	1.3	0.80–2.9	E
*L. scabrum* (caps)	3.3 ± 2.7–6.6 ± 3.2	2.4–5.7	0.42–14	F
*L. rufum* (caps)	0.36 ± 0.25–4.5 ± 5.6	0.30–1.6	0.08–21	G

A (Falandysz et al. 2008a, 2011; Frankowska et al. 2010; Zhang et al. 2010); B (Cocchi et al. 2006); C (Giannaccini et al. 2012); D (Jarzyńska et al. 2011; Kojta et al. 2011; Gucia et al. 2012a, b); D (Jarzyńska and Falandysz 2012a); E (Jarzyńska and Falandysz 2012b); F (Falandysz et al. 2007e; Zhang et al. 2013); G (J.F., unpublished)

^a^95 % confidence interval

Cultivated mushrooms such as *A. bisporus*, *P. ostreatus*, and *L. edodes* from Hungary showed Cd in caps of 0.17 ± 0.13, 0.91 ± 0.32, and 0.71 ± 0.48 mg kg^−1^ dm (Vetter et al. 2005), and this is at the lower edge of the average Cd concentrations noted in some wild-grown species (Table 9). The reported Cd contents of *A. bisporus*, *Agaricus* spp*.*, *P. ostreatus*, *P. florida*, *P. eryngii*, *P. salmoneo-stramineus*, and *L. edodes* on sale (or purchased) in Brazil are 0.011 to 0.23 mg kg^−1^ dm, and these are only weakly contaminated (Maihara et al. 2008). Surprisingly high concentration of Cd with an average value of 10 ± 1 mg kg^−1^ dm, which exceeds the European Union limit of 2.0 mg kg^−1^ dm by fivefold (see below), has been noted in the fruit bodies of commercially cultivated *Agaricus blazei* bought at a market in Kunming (Yunnan, China) (Sun et al. 2012). The PTWI set by the Joint FAO/WHO Expert Committee on Food Additives for Cd is 7 mg kg^−1^ bm (equivalent to 1 mg kg^−1^ bm per day) (from Gucia et al. 2012b), and a tolerable weekly intake (TWI) of 2.5 μg/kg bw (equivalent to 0.36 mg kg^−1^ bm per day) was established by EFSA (from Gucia et al. 2012b).

*M. procera* is a mushroom with a large size and its caps are highly valued by mushrooming fanciers. In one study, Cd intake resulting from the consumption of a 300- or 500-g portion of caps of *M. procera* was estimated as 4.7–111 and 24.5–185 μg (median Cd content between 0.049 and 0.37 mg kg^−1^ fw, assuming water content of 90 %), and these are equivalent to 0.078–1.85 and 0.41–3.1 μg kg^−1^ bm (for an adult of 60 kg bm) (Gucia et al. 2012b). It was concluded in this study by Gucia et al. (2012b) that in a good “mushroom” year, the consumption of large quantities of *M. procera* will lead to a short time (2 weeks to up to 2 months) exposure of fanciers to elevated Cd doses that exceeded the PTWI and TWI recommendations. Hence, Cd in caps of *M. procera* at some of the sites surveyed might be of concern to consumers, if eaten frequently by an individual in the mushrooming season.

Cadmium is among the minerals leached out of fruit bodies during boiling. The boiling and microwaving, with water, of *A. blazei* reduces its Cd content by 36 and 30 %, respectively (Sun et al. 2012). Accessibility of Cd from mushroom meals is more or less limited (cited after Gucia et al. 2012b). Accessibility of Cd from raw *A. blazei* will be greater (78 % by stomach and 69 % by gastrointestinal mimetic digestion) compared to when the mushrooms are boiled (51 and 46 %) and microwaved (58 and 50 %) (Sun et al. 2012). It should be noted that we do not recommend eating of any mushroom raw, and one of the reasons is agaritin, which is a phenylhydrazine derivative present in mushrooms of the genus *Agaricus* and that is hazardous to human health (Andersson and Gry 2004). Agaritin is deactivated when mushrooms are heat-cooked.

*Lead* as discussed earlier is excluded in mushrooms (BAF < 1), but this toxic metallic element compared to Cd or Hg is more abundant in the top layers of forest soils. For example, Pb in the top layer of soils beneath *B. edulis* and/or *M. procera* in the mountain areas of Tuscany of Italy was at 26 to 47 mg kg^−1^ dm (Giannaccini et al. 2012), at 6.7 to 37 mg kg^−1^ dm in Tucholskie forests of Poland (Jarzyńska et al. 2011), and 26 to 270 mg kg^−1^ dm in the top soil beneath *X. badius* at the forested mine dump site of a medieval gold and copper mine in Sudety mountains (Kojta et al. 2012). Hence, even if the potential to bioaccumulate Pb by species is low (BAF < 1), a quantity of Pb sequestered in the flesh by a small portion of mushrooms that emerged at “unpolluted” sites can ordinarily be of health concern (Table 10), but metal accessibility from this type of food is unknown. The maximum concentration of Pb permitted by the European Union regulation in cultivated *A. bisporus*, *P. ostreatus*, and *L. edodes* is 0.3 mg kg^−1^ fw (equivalent to 3.0 mg kg^−1^ dm, assuming 90 % moisture) (cited after Gucia et al. 2012b).

**Table 10:** Lead in mushrooms (adapted)

Species	Content (mg kg^−1^ dm)			Ref.
	Mean/s ± SD	Median/s	Total range	
*B. edulis* (caps)	0.51 ± 0.19–2.0 ± 0.9	0.22–0.94	0.10–2.9	A
*B. edulis* (whole)	1.2	–	0.37–2.1^a^	B
*B. edulis* (whole)	0.8 ± 0.3–2.6 ± 0.8	0.9–2.8	0.4–3.4	C
*B. edulis* (h/rfb)	0.70 ± 0.30/0.72 ± 0.30	–	–	D
*M. procera* (caps)	1.3 ± 0.5–8.5 ± 2.4	1.3–8.9	0.02–18	E
*M. procera* (whole)	3.4	–	2.2–4.7	B
*M. procera* (whole)	0.9 ± 0.3–10 ± 4	0.9–8.7	0.7–15	C
*M. procera* (h/rfb)	1.8 ± 1.6/1.2 ± 0.7	–	–	D
*L. pseudoscabrum* (caps)	0.53 ± 0.28	0.42	0.18–1.1	F
*L. duriusculum* (caps)	0.36 ± 0.14	0.35	0.14–0.69	G
*L. scabrum* (caps)	0.56 ± 0.23–4.1 ± 4.2	0.52–3.0	0.22–16	H
*L. scabrum* (h/rfb)	1.2 ± 0.5/1.4 ± 0.5	–	–	D
*L. rufum* (caps)	0.26 ± 0.16–1.2 ± 1.2	0.19–0.76	0.06–5.0	I

A (Falandysz et al. 2008a); B (Cocchi et al. 2006); C (Giannaccini et al. 2012); D (García et al. 2009); E (Jarzyńska et al. 2011; Kojta et al. 2011; Gucia et al. 2012a, b); F (Jarzyńska and Falandysz 2012a); G (Jarzyńska and Falandysz 2012b); H (Falandysz et al. 2007e; Zhang et al. 2013); I (J.F., unpublished)

*h* hynenophore, *rfb* rest of fruit body

^a^95 % confidence interval

In a recent study, the concentrations of Pb in hymenophore and the rest of the fruit body of such edible but probably not frequently eaten species such as *C. comatus*, *A. campestris*, and *Lepista nuda* were at 3.6 and 4.1, 3.0 and 2.2, and 2.5 and 2.3 mg kg^−1^ dm (no statistical difference between those morphological parts was noted) (García et al. 2009). In cultivated species, Pb concentrations were mostly low, below 3.0 mg kg^−1^ dm, and examples are data from Mexico where *A. bisporus* (caps) contained on average 0.41 mg kg^−1^ dm and *P. ostreatus* (whole) 0.91 mg kg^−1^ dm, while the spread of data for both species from reports on global scale is wide (Muñoz et al. 2005).

The acceptable daily intake of Pb for adults is between 0.21 and 0.25 mg per day and 1.5 and 1.75 mg per week (from Jarzyńska and Falandysz 2011a, b). In the case of *M. procera*, the estimated Pb intake from consumption of 300 or 500 g caps was 42–267 and 70–445 μg per meal, respectively. These values were based on the median concentrations of Pb found. It was concluded that, at some sites, the Pb intake rates show a cause for concern associated with Pb resulting from the consumption of between 300 and 500 g caps daily, on a frequent basis in the mushrooming season (Gucia et al. 2012b).

*Silver* is toxic to animal cells and Ag^+^ ion is highly toxic to bacteria. This is because Ag ion has a high affinity for sulfhydryl, amino, and phosphate groups, and it readily complexes with many endogenous ligands of the mammalian body. As given earlier, Ag at background and in geoanomalous and contaminated areas is very efficiently bioaccumulated by many mushrooms (Borovička et al. 2007, 2010b; Falandysz et al. 1994a, b; Falandysz and Danisiewicz 1995; Kojta et al. 2012). Data on Ag in several mushrooms from areas with Ag anomaly can be found in the article by Borovička et al. (2010b).

Apart from hyperaccumulators of this element (see above), it can be found at elevated contents in popular species, e.g., *M. procera* that were collected from background areas contained up to 4.8 mg kg^−1^ dm (median value) (Table 11). Silver was found also in cultivated *A. bisporus* (Table 11), but the number of available data for cultivated mushrooms seems to be very limited. To our knowledge, no data were published on the accessibility of Ag from mushrooms (cooked) to man, and the content of this metal in foods is not regulated.

**Table 11:** Silver in mushrooms (adapted)

Species	Content (mg kg^−1^ dm)			Ref.
	Mean/s ± SD	Median/s	Total range	
*B. edulis* (caps)	0.51 ± 0.19–2.0 ± 0.9	0.22–0.94	0.48–22	A
*B. edulis* (whole)	0.68 ± 0.94	–	0.16–2.6	B
*M. procera* (caps)	0.10 ± 0.22–5.5 ± 3.4	0.05–4.8	0.01–12	C
*L. pseudoscabrum* (caps)	0.57 ± 0.15	0.52	0.40–0.96	D
*L. duriusculum* (caps)	0.93 ± 0.24	0.86	0.58–1.4	E
*L. scabrum* (caps)	0.47 ± 0.64–0.76 ± 0.35	0.45–0.78	0.12–1.9	H
*L. rufum* (caps)	0.12 ± 0.04–0.93 ± 0.35	0.11–0.93	0.03–1.6	I
*L. versipelle*	0.27 ± 0.03	–	0.22–0.29	J
*L. vulpinum*	2.0 ± 0.6	–	1.5–3.1	J
*A. bisporus* (caps)	0.55 ± 0.14	–	0.42–0.79	K
*A. bisporus* (whole)	0.30 ± 0.07–0.46 ± 0.09	–	0.15–0.58	K

A (Falandysz et al. 1994a; 2008a); B (Falandysz et al. 1994a); C (Falandysz et al. 2008b; Jarzyńska et al. 2011; Kojta et al. 2011; Gucia et al. 2012a, b); D (Jarzyńska and Falandysz 2012a); E (Jarzyńska and Falandysz 2012b); H (Falandysz et al. 1994a, 2007e; Zhang et al. 2013); I (Falandysz et al. 1994a; J.F., unpublished); J (Falandysz et al. 1994a); K (Falandysz et al. 1994b)

## Radioactivity of mushrooms

Wild-grown mushrooms in several reports are described as biota that are efficient accumulators and bioindicators of environmental diffusion of radionuclides. Emphasis is frequently given on long-lived ^137^Cs that polluted the surface of the Earth due to global radioactive fallout after testing of nuclear weapons and from nuclear power plant accidents, but some others as well as naturally occurring radionuclides are also intensively studied in mushrooms (Horyna and Řanda 1988; Bakken and Olsen 1990; Tsvetnova and Shcheglov 1994; Dahlberg et al. 1997; Kirchner and Daillant 1998; Rühm et al. 1997; Skwarzec and Jakusik 2003; Strandberg 2004; Baeza et al. 2004a, b; Kostiainen 2007; Vaartamaa et al. 2009; Mietelski et al. 2002, 2010; Taira et al. 2011; Vinichuk et al. 2011; Castro et al. 2012; and many others).

The capacity of mushrooms to sequester radioactive elements in fruit bodies and so the rates of their uptake vary between species, and good examples to illustrate this are data on the contents of the most studied—^137^Cs. There is no reason for mushrooms to discriminate between stable and radioactive isotopes of elements when absorbed by the mycelium and accumulated in the mushroom flesh, but their accessibility at sites where the mycelium lives can be different. There are several types of radioactivity, and the decay of elements results in the emission of extremely toxic subatomic particles such as alpha (α) particle (helium nuclei, i.e., two protons, He^2+^) and less potent beta minus (energetic electrons, *β* ^−^) and beta plus (β^+^, positrons) particles and also of gamma (γ) ray (photon) with nuclide’s half-life (*T*_1/2_) equivalent respectively from several days to millions of years (Skwarzec 2012).

Some of the radioactive elements naturally occurring in nature that are the primordial non-series radionuclides of terrestrial (e.g., ^40^K and ^87^Rb) and cosmogenic origin (e.g., ^3^H, ^7^Be, ^14^C, ^22^Na) and that of uranium (^238^U), thorium (^232^Th), actino-uranium (^235^U), and neptunium (^237^ Np) decay series have been found in mushrooms, but published data are few (see below). The cosmogenic ^3^H, ^7^Be, ^14^C, and ^22^Na are among the most important radionuclides because of their long half-life (*T*_1/2_) equivalent to 12.33 years for ^3^H, 53 days for ^7^Be, 5,740 years for ^14^C, and 2.6 years for ^22^Na, and they are of *β* ^−^ (^3^H, ^14^C) and β^+^ (^22^Na) radioactivity, and ^7^Be is capturing electrons. These cosmogenic radionuclides in foods are without radiotoxic importance because they occur at an extremely small concentration in nature (Skwarzec 2012).

The decay series radionuclides are ^238^U (*T*_1/2_ of 4,500 million years; α), ^232^Th (*T*_1/2_ of 14,100 million years; α), ^235^U (*T*_1/2_ of 704 million years; α), and ^237^ Np (*T*_1/2_ of 2.14 million years; α) series that during radioactive decay transform into many other radioactive elements, which can be, respectively, the emitters of α and β^−^ particles. And apart from ^238^U, ^232^Th, ^235^U, and ^237^ Np, the most risky in foods are ^234^U (*T*_1/2_ of 245,500 years; α) and ^230^Th (*T*_1/2_ of 75,380 years; α), while much weaker source of radiation dose are ^231^ Pa (*T*_1/2_ of 32,760 years; α), ^228^Th (*T*_1/2_ of 1.9 years; α), ^229^Th (*T*_1/2_ of 734 years; α), ^227^Th (*T*_1/2_ of 18.7 days; α), ^225^Ac (*T*_1/2_ of 10 days; α), ^223^U (*T*_1/2_ of 159,200 years; α), and ^210^Po (*T*_1/2_ of 138 days; α). Data on these types of element in mushrooms are almost non-existing and the exception is U for which many details can be found in the article by Gadd and Fomina (2011).

Since the 1940s, many radionuclides of anthropogenic origin have been released into the environment, and their sources relate to the testing of nuclear weapons, operation of nuclear power plants, radioactive waste disposal, and the manufacture of radioactive compounds. They are categorized into three groups: neutron activation products (1), fission products (2), and transuranic elements (3) (Skwarzec 2012).

The activation of stable isotopes by neutron irradiation can produce various radionuclides, and their major source in nature is nuclear weapon testing, nuclear reprocessing, nuclear power plants, and nuclear research. By using modern radioanalytical methods, it is possible to detect the activation products such as ^22^Na, ^51^Cr, ^54^Mn, ^65^Zn, ^110m^Ag, and ^124^Sb. The most important, due to its strong radioactivity and long-life, of the neutron activation (1) products are radionuclides such as ^54^Mn (*T*_1/2_ of 312 days; *electrons capturing*), ^55^Fe (*T*_1/2_ of 2.737 years; *electrons capturing*), ^60^Co (*T*_1/2_ of 5.2714 years; *β* ^−^, γ), ^63^Ni (*T*_1/2_ of 100.1 years; *β* ^−^), ^64^Cu (*T*_1/2_ of 12.7 days; β^+^), ^110m^Ag (*T*_1/2_ of 249.9 days; *β* ^−^), ^124^Sb (*T*_1/2_ of 60.2 days; *β* ^−^), and ^125^Sb (*T*_1/2_ of 2.76 years; β^−^). Some of these radionuclides have been reported in mushrooms (Gentili et al. 1991).

The most important due to strong radioactivity and long-life, the radioactive fission products (2) formed in the explosion of nuclear bombs, both the uranium and plutonium bombs are radionuclides such as: ^90^Sr (*T*_1/2_ of 28.79 years; β^−^), ^131^I (*T*_1/2_ of 8.02 days; β^−^ and γ) and ^137^Cs (long-lived, *T*_1/2_ of 30.17 years. As indicated earlier, ^137^Cs in mushrooms is well documented and data on both ^137^Cs and ^134^Cs, which have different half life-time, help to study sources of pollution of mushrooms with those nuclides and also diffusion rates in soils (Mietelski et al. 2010).

For explosions in the atmosphere, the depositions of those radionuclides reaching the earth are 78 % from stratospheric, 12 % from local, and 10 % from the tropospheric fallout (Skwarzec 2012). Radiocesium (^137^Cs) is the most relevant nuclide from this source in mushrooms (see below). Also disasters of nuclear power plants apart from heavy local pollution are relevant regional sources of those radionuclides and largely of ^137^Cs to soils, plants, mushrooms, and other biota in the food chain, e.g., the Chernobyl accident in 1986 (Dahlberg et al. 1997; Kostiainen 2007; Mietelski et al. 2010) and the Fukushima accident in 2011.

The transuranic elements (3) all are radioactive. Nuclear weapon tests and nuclear power production are major sources of transuranic elements to ambient environment (Np, Pu, Am, and Cm).

From the point of view of strong radioactivity and long-life time, the most important among naturally occurring radionuclides mentioned are ^40^K, ^87^Rb, and some of the ^238^U, ^232^Th, ^235^U, and ^237^ Np decay series. Radon from ^238^U decay series that emanates from the ground into the atmosphere decays to ^210^Pb (*T*_1/2_ 22 years; *β* ^−^) and ^210^Po (*T*_1/2_ 138 days; α), and both return to the earth via wet deposition and on particles as dry deposition. The decay of ^238^U also produces ^210^Pb and ^210^Po in soil that are referred to as “supported” ^210^Pb and ^210^Po. Complementary sources of atmospheric ^210^Po include dust storms, coal-burning, forest fires, and plant exudates (Skwarzec 2012). Both ^210^Po and ^210^Pb are found in mushrooms (Mietelski et al. 2002; Skwarzec and Jakusik 2003; Baeza et al. 2004a).

Artificial radionuclides with ^137^Cs as the major nuclide can be found in any wild-grown mushroom due to its deposition from the global radioactive fallout and persistency, while concentrations in mushrooms vary between species and regions and also on time scale. And one of the reasons is that with time passing, the radionuclides also undergo the process of infiltration (diffusion from surface down) into deeper soil layers. This process can be very slow. Mushrooms can capture ^137^Cs from the surface of soils and decaying litter and from deeper soil layers, and this is related to fungal biology and placement of mycelium and depth and structure of soil layers. Fungi certainly are involved in biogeocycling of ^137^Cs and by decaying fruit bodies—like in the case of other mineral elements, fungus can recycle ^137^Cs from deeper soil layers back to the surface of forest carpet (Lepp et al. 1987; Smits and Hoffland 2009; Gryndler et al. 2012). More or less severe cases of mushroom contamination with artificial radionuclides have been reported at sites or regions of nuclear weapons testing and nuclear power plant failure (Taira et al. 2011).

## Potassium and radiopotassium

The terrestrial origin of ^40^K (*T*_1/2_ of 1.248 × 10^9^ years; β^−^, electrons capturing) and ^87^Rb (*T*_1/2_ of 4.92 × 10^10^ years; emits *β* ^−^) is a significant source of radiation to humans while both occur at low concentrations in the rock crust (<1 mBq kg^−1^) (Skwarzec 2012). Mushrooms are rich in K and Rb (Tables 1 and 12). Typical contents of K and Rb in mushrooms are around 25,000 to 50,000 and ~100 to 300 mg kg^−1^ dm, respectively (Table 12). And fruit bodies are at least one order of magnitude richer in K and Rb and also in Cs compared to fungal mycelium (Vinichuk et al. 2011). The radionuclides ^40^K and ^87^Rb are common constituents of mushrooms (Baeza et al. 2004a, b, 2005; Mietelski et al. 2010; and many others). The contents of ^40^K in mushrooms and other foods can be assessed from the concentrations of total K (by multiplying with 0.000117). Rubidium in nature consists of two isotopes, a stable ^85^Rb (72.2 %) and radioactive ^87^Rb (27.8 %), and no data published are available on ^87^Rb in mushrooms.

**Table 12:** Alkali metals and radionuclides (Na, K, Rb, Cs, ^137^Cs, and ^40^K) in several species of mushrooms (data on Na, K, Rb, and Cs are for caps of fruit bodies collected in ~1986 ~ 2006 and data on ^137^Cs and ^40^K are for whole fruiting bodies) collected across of Poland (mean or median values and their range, respectively; adapted)

Species	Na (mg kg^−1^ dm)	K (mg kg^−1^ dm)	Rb (mg kg^−1^ dm)	Cs (mg kg^−1^ dm)	^137^Cs (Bq kg^−1^ dm)	^40^K (Bq kg^−1^ dm)	Ref.
*S. luteus*	55	39,000	–	20 ± 5	12,000^a^	2,200^a^; *Suillus grevillei*	A
*B. edulis*	170–200	24,000–25,000	330–370	1.6–2.3			B
*B. edulis*	100^b^	26,000^b^	650^b^	8.7^b^	5,100–13,000^a^	970–2,100^a^	B
*L. amethystina*	360	51,000	–	0.79	5,600^a^	2,100^a^	A
*T. equestre*	60	70,000	–	9.5 ± 2.6			C
*T. equestre*	200–4,200	32,000–83,000	730–1,300		10,000^a^	3,400^a^; *Tricholoma terrerum*	D
*A. solidipes* ^c^	70	53,000		0.95	315^a^	1,800^a^	A
*L. scabrum*	270–380	34,000–52,000	240–330	2.7–6.0			E
*L. scabrum*	290^b^	35,000^b^	320^b^	7.2^b^			E
*M. procera*	20–290	25,000–47,000	18–230	0.015–0.043	1,100^a^	1,500^a^	F
*P. involutus*	90–520	30,000–63,000	130–670	0.8–7.8			F
*P. involutus*	180^b^	30,000	410^b^	20^b^	2,500–10,000^a^	2,000–2,300^a^	F
*A. muscaria*	30–37	37,000–45,000	58–300	0.063–0.83			F

A (Falandysz et al. 2001a, b; Mietelski et al. 2010); B (Falandysz et al. 2008a); C (Falandysz et al. 2001a, b; J.F., unpublished); D (Mietelski et al. 2010; J.F., unpublished); E (Falandysz et al. 2007e); F (Falandysz et al. 2008b; Mietelski et al. 2010; Jarzyńska et al. 2011; Kojta et al. 2011; Drewnowska et al. 2012c; Gucia et al. 2012a, b); G (Falandysz et al. 2007d; Brzostowski et al. 2009, 2011a, b; Mietelski et al. 2010); H (Falandysz et al. 2007f)

^a^A “hot spot” site near the city of Opole in south-western Poland in 2006–2007

^b^Data for specimens collected from Kłodzka Dale in Sudety Mountains in south-western Poland

^c^Earlier called also as *A. mellea* and *A. ostoyae*

The activities of ^137^Cs in fruit bodies were often compared with the natural content of ^40^K. Potassium as an essential nutrient is “bio-adjustable” in that for a given species a certain concentration of K is favorable, and thus, the activity of ^40^K is relatively constant and this is typical for the given species and site.

## Cesium and radiocesium

Radiocesium (^137^Cs) is the nuclide on which public interest is mainly focused and it is widely studied in wild-grown mushrooms (Kirchner and Daillant 1998; Baeza et al. 2004b, 2005; Mietelski et al. 2002, 2010; Kostiainen 2007; Taira et al. 2011; Castro et al. 2012; and many others). The ^137^Cs and other nuclides deposited from fallout are further absorbed by fungi from soils (decaying litter and organic and inorganic soil layers—elements diffused into soil solution), and there is no discrimination between decaying and stable isotopes of a particular element undergoing uptake by mycelia. Hence, the efficiency of radionuclides bioaccumulation in mushrooms as a phenomenon does not vary from that of metals at sites polluted due to depositions from smelters: that which is easily accessible is readily accumulated and no extra energy is lost by the organism. A good example to explain this are metals (e.g., Ni and Cd) found at elevated concentrations in mushrooms collected at sites exposed to metal fumes deposited from metal smelters (Barcan et al. 1998; Collin-Hansen et al. 2002). “Efficient” bioaccumulation of ^137^Cs by fungi is a feature related to mushroom species which depends on the “need” for stable Cs, which is an abundant element in mushrooms (Table 12) and on the efficiency of radionuclide diffusion into soil solution (stable Cs can be supplied from other pools which can be less accessible and to deficiency of ^137^Cs in solution).

A large-scale remediation of soils polluted with radionuclides and especially with ^137^Cs is out of real possibilities and polluted sites become abandoned grounds for years. Rosén et al. (2011) suggested that a single (100 kg K/ha) treatment of forest soil with potassium (KCl) fertilizer in 1992 could have a long-term (even 17 years after application) impact on bioaccumulation of ^137^Cs by *Cortinarius semisanguineus*, *Lactarius rufus*, *Cortinarius caperatus*, and *S. variegatus* mushrooms—radioactivity is reduced from 21 to 58 %. An attempt to use stable Cs salts to reduce uptake of ^137^Cs by forest mushrooms and berries has not been reported.

Mushroom contents of stable Cs vary between its morphological parts, and in caps, it is around twice more abundant in Cs than in stipe. High geographical (spatial) variations in the contents of Cs in mushrooms have been noted. For example, the mycorrhizal species such as *B. edulis* and *L. scabrum* with 1.6 to 8.7 mg Cs kg^−1^ dm (median values) in caps are richer in this element compared to mycorrhizals, such as *A. muscaria* with 0.063 to 0.83 mg kg^−1^ dm and up to 20 mg kg^−1^ dm in *P. involutus* (Falandysz et al. 2007c, d, e, 2008a). The saprophytic *M. procera* contained stable Cs in caps at 0.015 to 0.043 mg kg^−1^ dm (median values) (Falandysz et al. 2008b), and this is low compared to mycorrhizal mushrooms mentioned. Hence, there is no doubt that ^137^Cs can be more efficiently sequestered in flesh by many mycorrhizal mushrooms than saprophytic mushrooms (Rühm et al. 1997).

As mentioned, the largest radioactivity from artificial radionuclides in mushrooms usually comes from ^137^Cs, but this can differ regionally. In a study in France, the contribution to the gamma-radioactivity from ^134^Cs, ^137^Cs, ^210^Pb, and ^226^Ra for the majority of mushrooms was highest for ^210^Pb. Mushrooms there contained ^210^Pb at <1.76 to 36.5 Bq kg^−1^ dm and for ^226^Ra was an order of magnitude less. And in species that are eaten less frequently and at lower rate, the radioactivity from ^137^Cs was greatest—up to 2,860 Bq kg^−1^ dm (Kirchner and Daillant 1998).

In studies in Finland, the activity concentrations of ^137^Cs in 600 samples of mushrooms collected in 2000–2005 were between 10 and 3,000 Bq kg^−1^ (low in *Leccinum* spp., *Gyromitra* spp., and *Scutiger ovinus*; medium in *B. edulis*, *C. cibarius*, and *Russula* sp.; and high in, e.g., *Cantharellus tubaeformis*, *C. cornucopioides*, *Lactarius* spp., *Hydnum* spp., *S. variegates*, and *Cortinarius caperatus*), and no significant reduction in activity could be noted over that time (Kostiainen 2007). In addition, after comparison of data given for radioactivity in the same species but collected at the same site in 1986–1988, no decrease was found from 1986 to 2005. For *B. edulis*, whose mycelium lives relatively deeper in soil layer, the radioactivity increased as the post-Chernobyl ^137^Cs infiltrated deeper into the soil horizon (Kostiainen 2007). Later, a similar result was noted for *B. edulis* elsewhere (Mietelski et al. 2010).

## Other radionuclides

Compared to ^137^Cs and ^40^K, less data are published on other radionuclides in mushrooms both natural and artificial. Other radionuclides studied in mushrooms include ^210^Po, ^234,238^U, ^228,230,232^Th, ^90^Sr ^239+240^Pu, ^238^Pu ^241^Am, ^134^Cs, ^85^Sr, ^60^Co and ^226^Ra (Kirchner and Daillant 1998; Mietelski et al. 2002; Skwarzec and Jakusik 2003; Baeza et al. 2004a; Szántó et al. 2007; Vaartamaa et al. 2009). In view of the current knowledge, the amounts of these radionuclides reported for edible wild-grown mushrooms that emerged at areas not close to sites of nuclear tests and accidents, as well as for cultivated species and mushrooms, are below accepted doses for internal exposure.

## Technical problems related to trace minerals in mushrooms

The truth is that accurate and precise analytical methods are needed when studying any mineral constituent in any abiotic or biotic matrices. In several articles (old and new) on trace minerals in mushrooms, unreliable data were published on elements (Se, Hg, Fe, Co, Cr, V, Ni, Rb, Ba, Cs, Sr, U, Zr and on the rare earth elements such as Ga, Nb, Ce, Th, Nd, and also Li, Cd, and Pb) sequestered in fruit bodies. Regrettably, many of such “dubious” data were published in journals that specialized in trace element research. A clear documentation of all such cases will need further investigations, and some aspects of such cases are discussed in detail elsewhere (Falandysz and Lipka 2006; Borovička and Řanda 2007; Falandysz 2008, 2012, 2013; Falandysz et al. 2012a, 2013; Borovička et al. 2011; Jarzyńska and Falandysz 2011a, b; Jarzyńska et al. 2012a). The biased analytical data published give a false picture of the composition and nutritional value of mushrooms with respect to minerals. Certainly, if a well-studied species is not classified as a hyperaccumulator of given mineral elements and is not collected from a polluted or geoanomalous site, a high load of element in its fruit body (example is Se, Hg, Fe, Cr, Co, Li, Cd, V, Ni, Ba, Sr, Zr) is not expected. When species of mushroom is without earlier published data on its mineral composition, the proper choice of analytical method and basic rules of chemical analysis must be applied.

A good example of this kind of problems is the case of Se (total Se) in mushrooms. As discussed earlier (see Se section), mushrooms have high variability in their contents of Se. Some mushrooms are specifically rich in this element and a credible range for all species studied until now is from ~0.01 to 370 mg kg^−1^ dm (some recent and totally biased data showed even around 190 to 420 mg Se kg^−1^ dm as typical values for certain species) (Falandysz 2013). Selenium for many reasons remains a challenge to analysts, agronomists, nutritionists, physicians, and the public. As mentioned, Se has a high biological potential and its content in foods is usually small, and also small Se doses are required in human nutrition usually between 1 and 3.3  μg kg^−1^ bm daily.

**Fig. 2:**
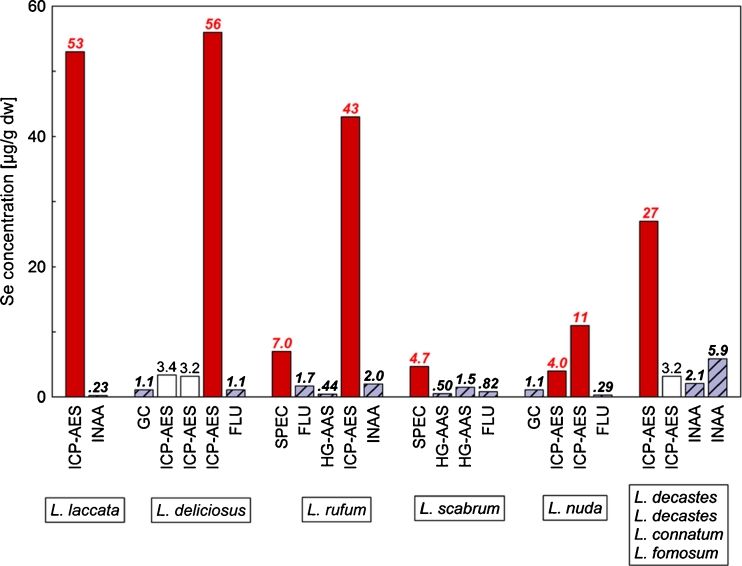
Selenium in mushrooms such as *Laccaria laccata*, *Lactarius deliciosus*, *Leccinum rufum*, *Leccinum scabrum*, *Lepista nuda*, *Lyophylum decastes*, *Lyophylum connatum*, and *Lyophylum fomosum* by several authors and techniques (mean values—data adapted from the references cited, respectively; color figure available online) [*dark shadowed bars* (in *red* online) relate to suspicious results because of highly excessive values reported due to selection of improper method of determination; the *empty bars* (in *white* online) relate to methods of measurement which can give incorrect result due to low sensitivity or nonspecific interferences that are difficult to control; and the *light shadowed bars with askew lines* (*shadowed in bluish* online) are data that appear to be acceptable and by valid methods] (from Falandysz 2013)

**Fig. 3:**
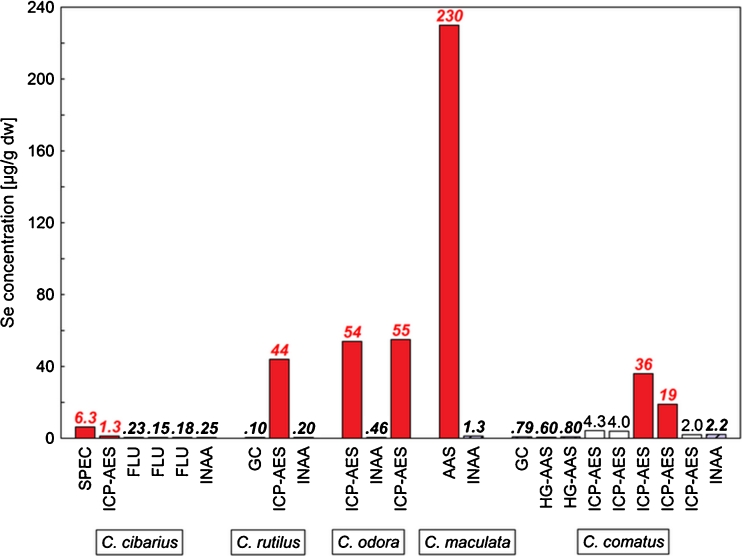
Selenium in mushrooms such as *Cantharellus cibarius*, *Chroogompus rutilus*, *Clitocybe odora*, *Collybia meculata*, and *Coprinus comatus* by several authors and techniques (mean values—adapted; color figure available online) (from Falandysz 2013)

Several of the sample preparation methods and instrumental measurement techniques have been applied for the examination of Se in mushrooms. These instrumental techniques include UV–VIS spectrophotometry, gas chromatography, flame atomic-absorption spectroscopy (AAS), hydride generation-atomic absorption spectroscopy (HG-AAS), hydride generation graphite furnace-atomic absorption spectroscopy (HG-GF-AAS), inductively coupled plasma atomic emission spectroscopy (ICP-AES), inductively coupled plasma mass spectrometry, fluorimetry (FLU), and neutron activation analysis (INAA) (Falandysz 2013). To determine total Se content of mushroom after wet digestion (with oxidizing acids), the Se contained in analyte needs to be separated (to enhance sensitivity and remove interferences) before aspiration to flame AAS. This is offered by HG-AAS and HG-GF-AAS but not by ordinary F-AAS because it lacks sensitivity for Se (Figs. 2 and 3). Also in the case of ICP-AES, the Se contained in the wet digested mushroom sample has to be separated (as hydride) before aspiration of digest to plasma. Determination of Se by ICP-AES without its pre-separation suffers due to common, nonspectral interferences, because of the formation of carbonyl ions that interfere with Se spectral line at *λ* 194.163 nm and this is a well-known fact (Machat et al. 2002). In several reports are given data on Se in mushrooms after determination by ICP-AES but without pre-separation of Se before instrumental measurement (Figs. 2 and 3).

## Electronic supplementary material

Below is the link to the electronic supplementary material.

ESM 1 (DOC 23676 kb)
