# Supplementary material for: Macro and trace mineral constituents and radionuclides in mushrooms: health benefits and risks
Source: Appl Microbiol Biotechnol. 2012 Nov 25;97(2):477–501. doi: 10.1007/s00253-012-4552-8 (PMC3546300; doi:10.1007/s00253-012-4552-8)
Supplement: Supplementary file 1 — (DOC 23676 kb) [file 253_2012_4552_MOESM1_ESM.doc]

Supporting materials available on-line

**Macro and trace mineral constituents and radionuclides in mushrooms – benefits, risks and problems**

Jerzy Falandysz^1*^ and Jan Borovička

^1^ Institute of Environmental Sciences & Public Health, University of Gdańsk, Gdańsk, Poland

^2^ Institute of Geology, Academy of Sciences of the Czech Republic, Prague, Czech Republic

^3^ Nuclear Physics Institute, Academy of Sciences of the Czech Republic, Prague, Czech Republic

^*^Correspondence: [jfalandy@chem.univ.gda.pl](mailto:jfalandy@chem.univ.gda.pl)

*************************************


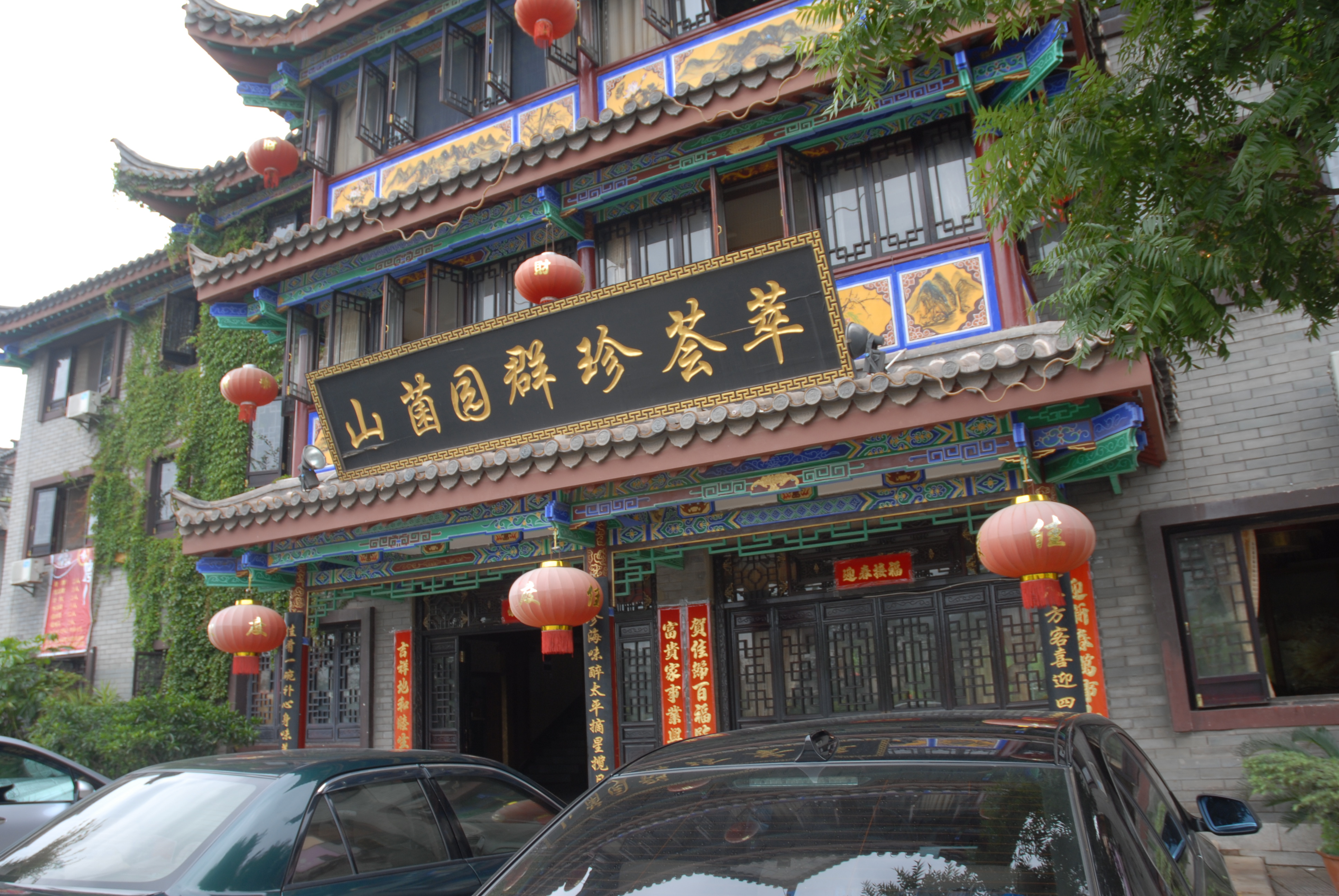


Fig. S1. The "Mountain mushrooms restaurant: delicacies gather together" in Chuxiong city of the Chuxiong Yi Autonomous Prefecture in Yunnan, China (by Jerzy Falandysz).


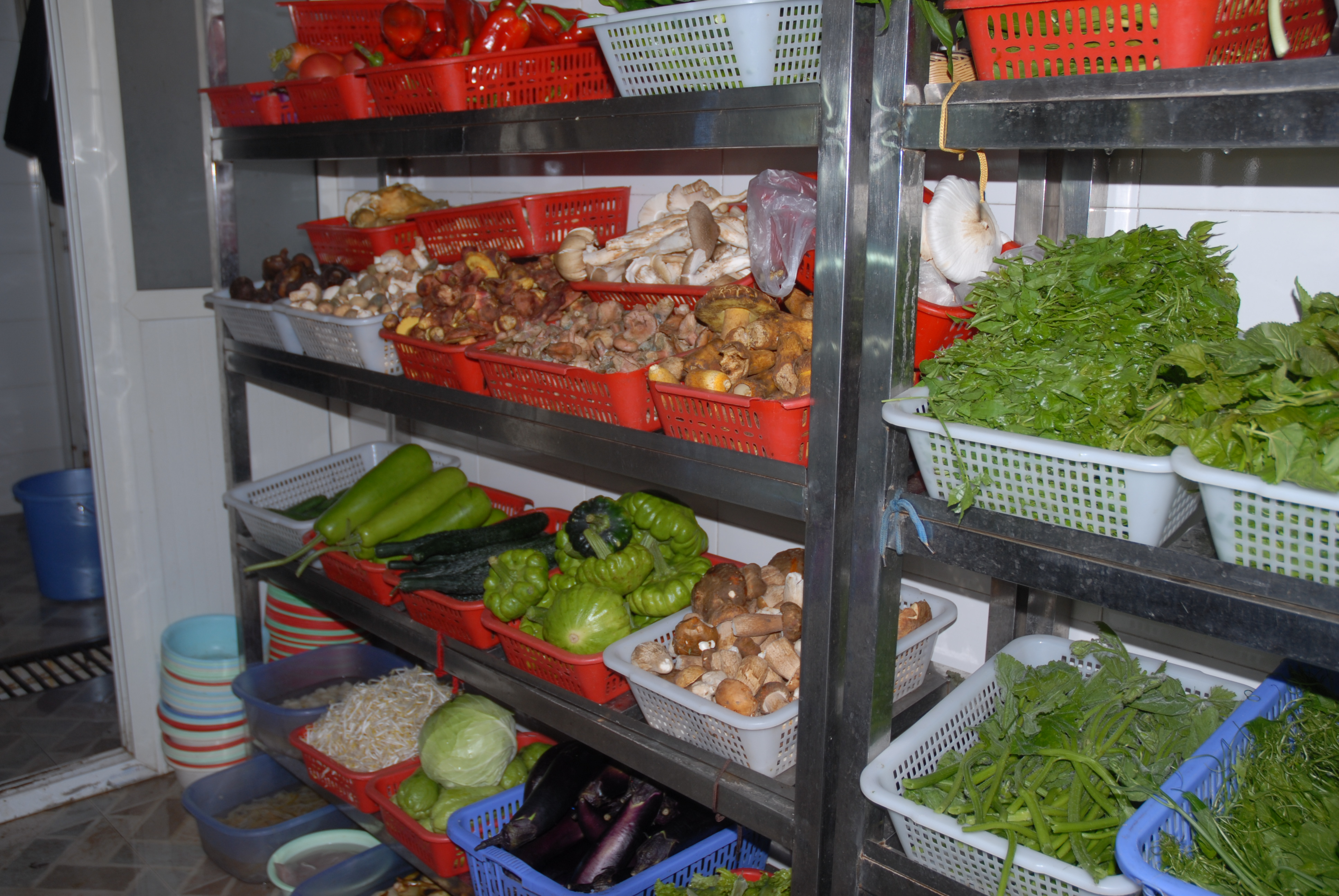


Fig. S2. Fresh supply of wild grown mushrooms (several *Boletus* spp. and *Tricholoma matsutake*) available to customers in "Mountain mushrooms restaurant: delicacies gather together" in Chuxiong city in Yunnan, China (by Jerzy Falandysz).


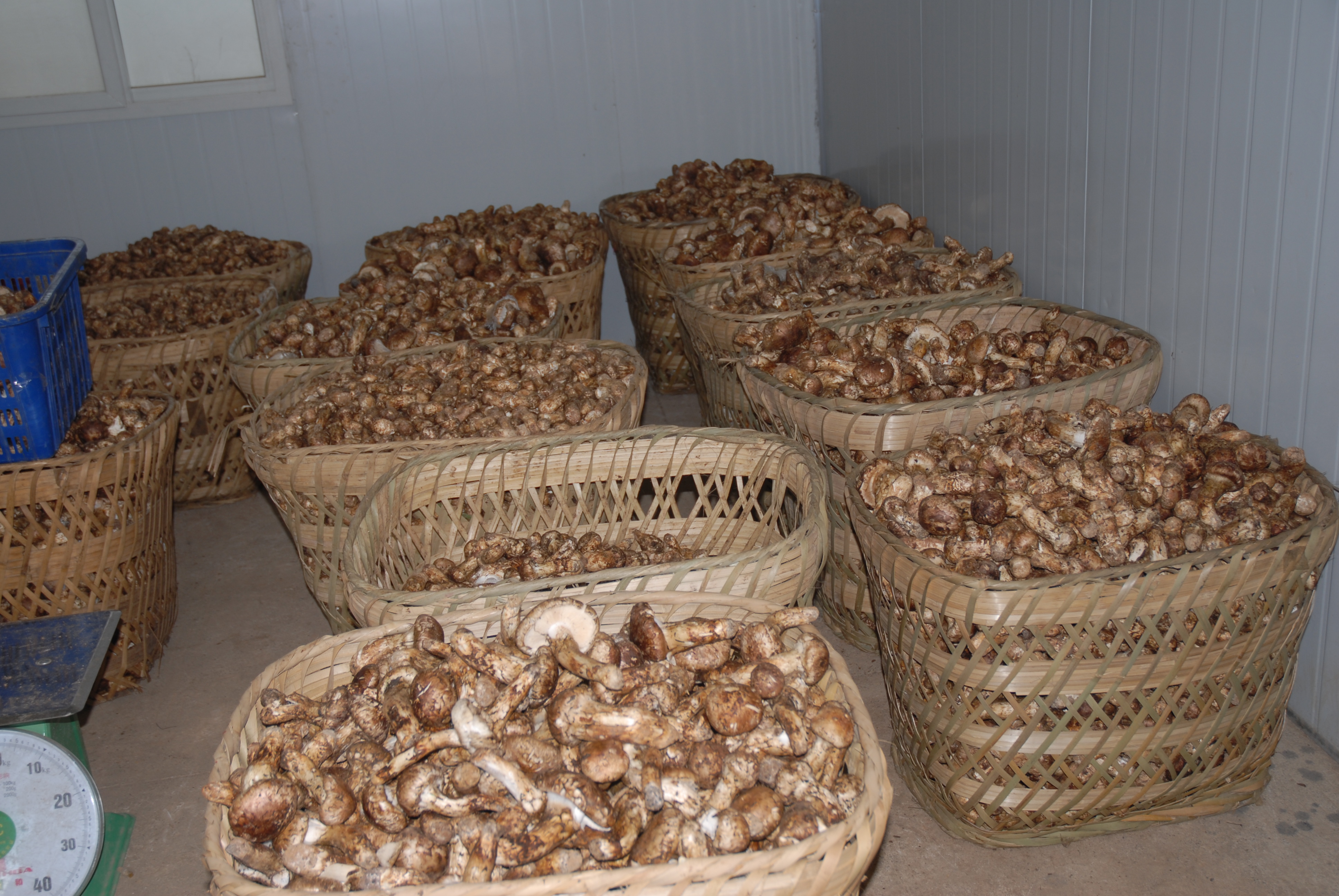


Fig. S3*. Tricholoma matsutake* prepared for sale at the mushrooms’ market in Shangri-la, Yunnan, China. Within the mushroom season the daily volume of wild grown mushrooms sold at the Shangri-la’s mushrooms market to other parts of China and for export abroad is *circa* 30 tons (by Jerzy Falandysz).


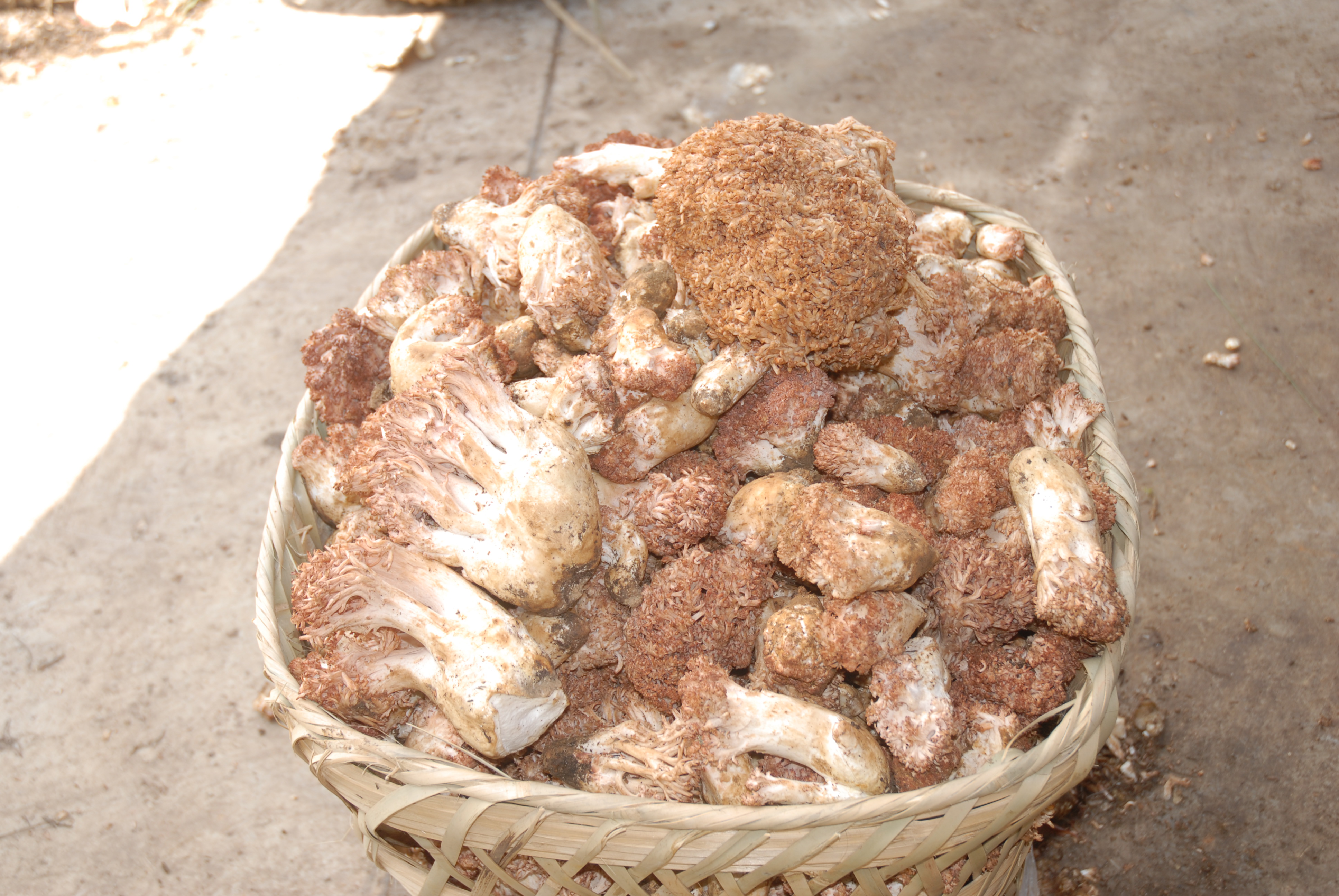


Fig. S4. Mushrooms *Ramaria rufescens* prepared for sale at the mushrooms’ market in Shangri-la, Yunnan, China (by Jerzy Falandysz).


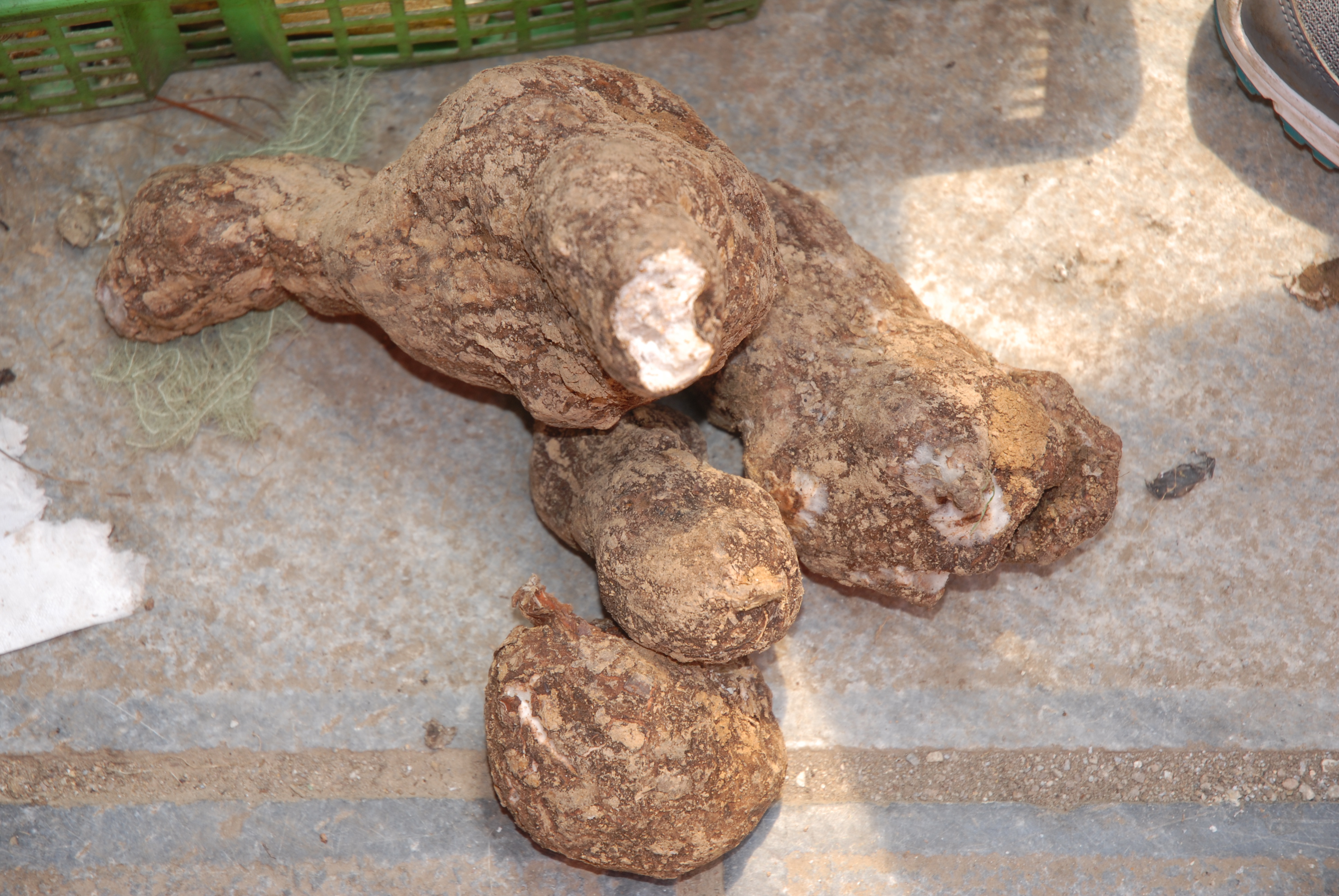


Fig. S5. Sclerotia of *Wolfiporia extensa* (Peck) Ginns in a shop at Baohe,

Weixi Lisu Autonomous County, Yunnan, China (by Jerzy. Falandysz).


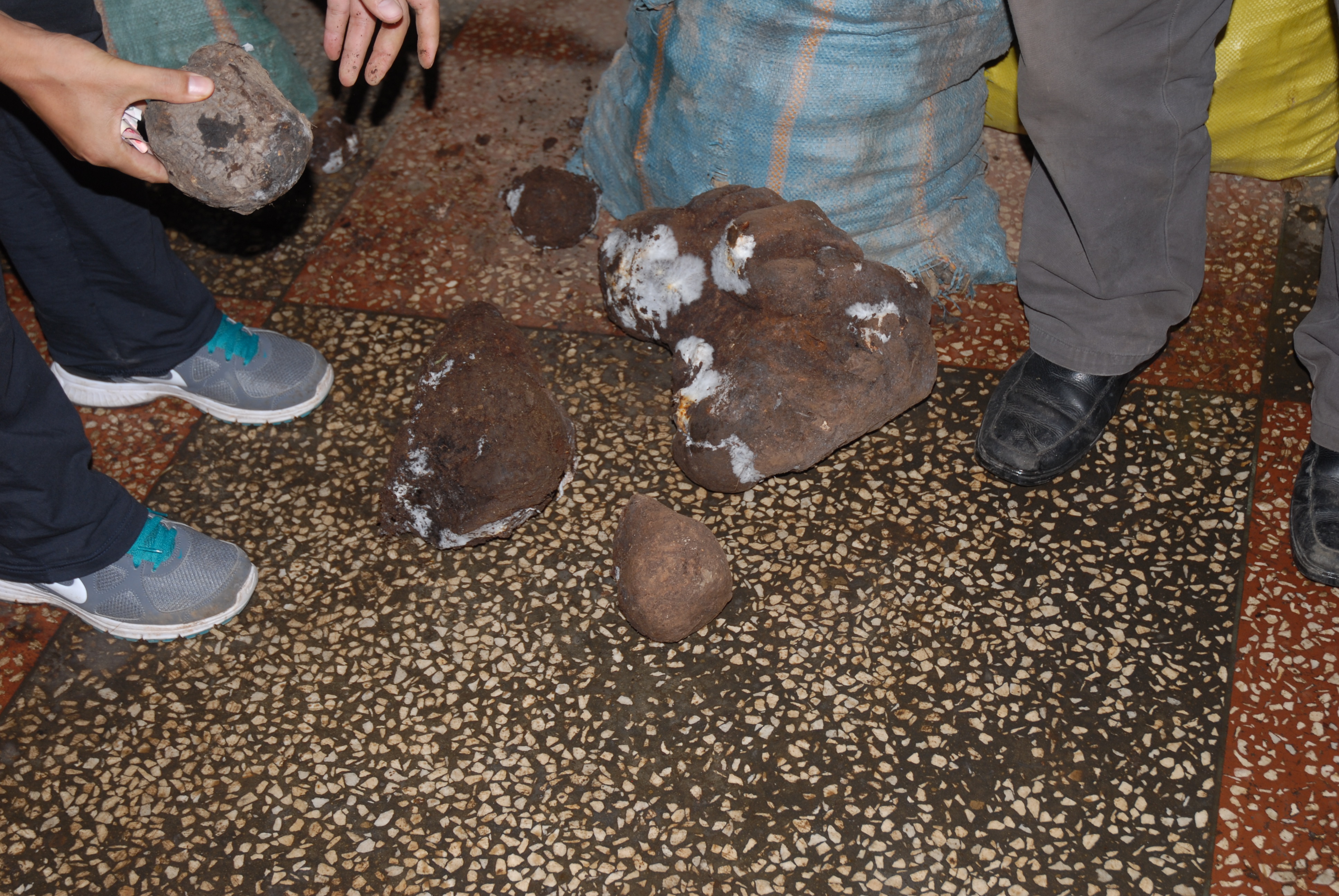


Fig. S6. Sclerotia of *Wolfiporia extensa* (Peck) Ginns (formerly known as *W*. *cocos* F.A. Wolf and *Poria cocos*) in a shop at Baohe, Weixi Lisu Autonomous County, Yunnan, China (by Jerzy Falandysz).


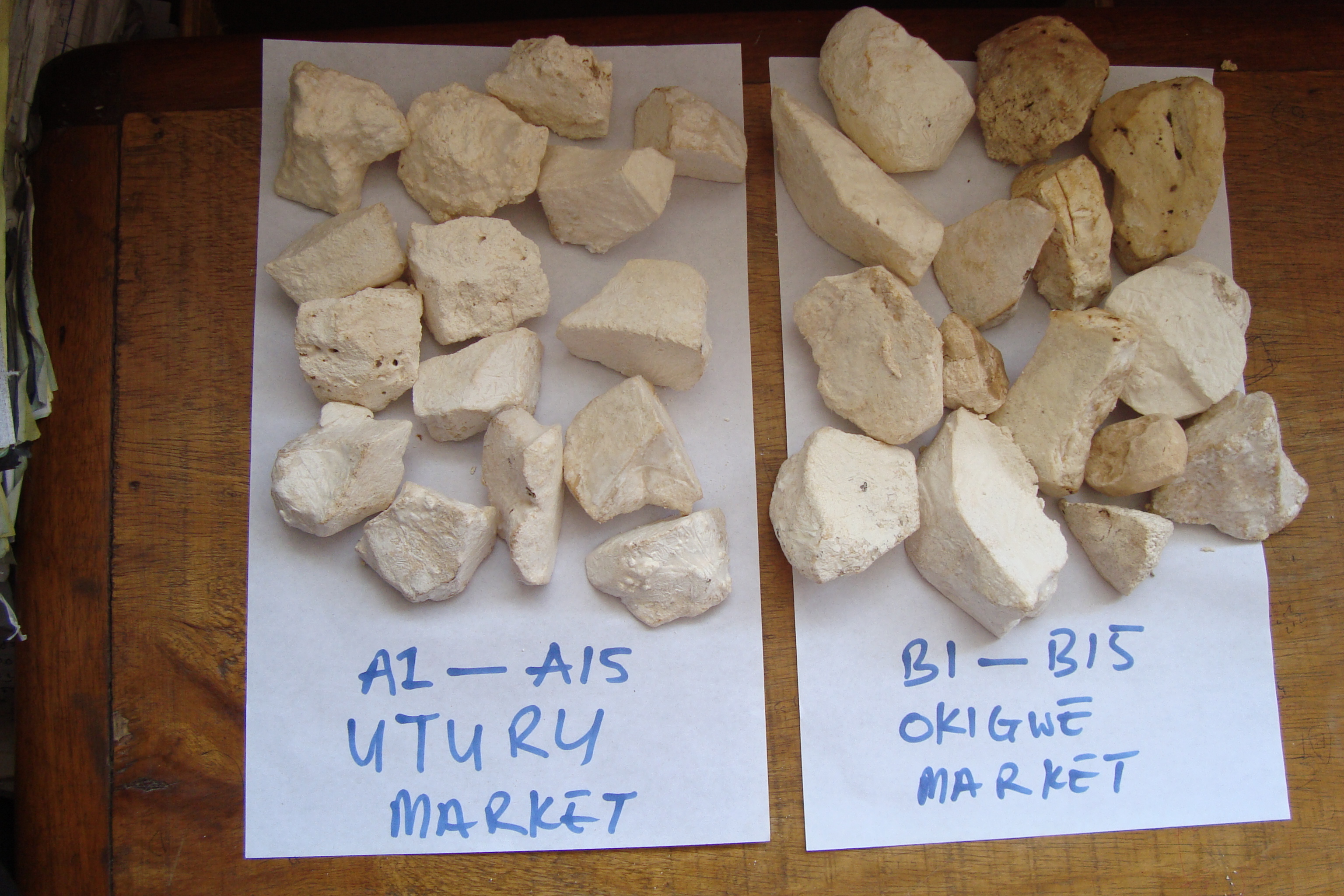
F Fig. S7. Sclerotia of *Pleurotus tuber-regium* (Abia State, Nigeria; by Innocent C. Nnorom).


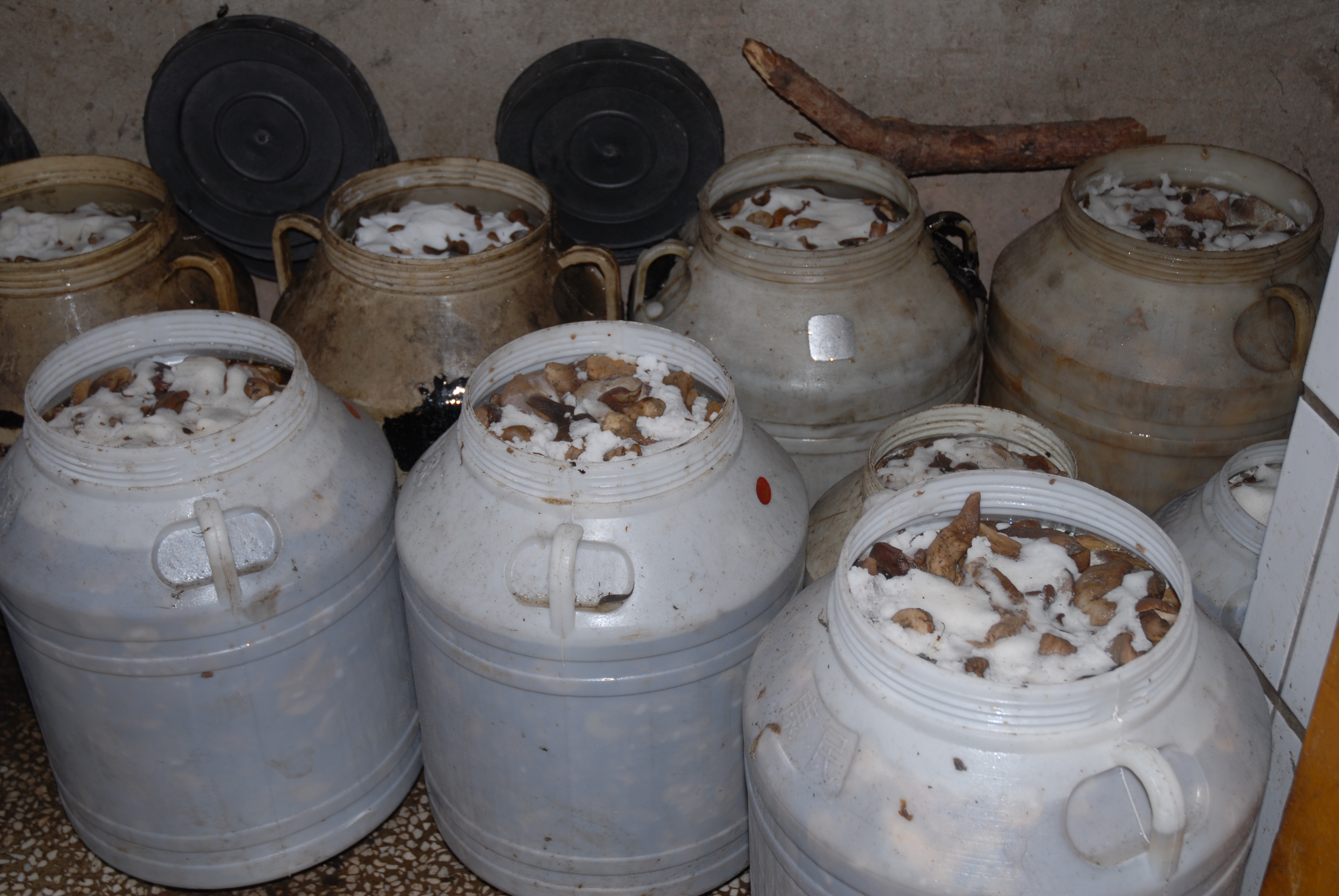


Fig. S8*.* Salting of *Boletus* and other mushrooms in a mushroom merchants shop at Baohe, Baohe, Weixi Lisu Autonomous County, Yunnan, China (by Jerzy Falandysz).


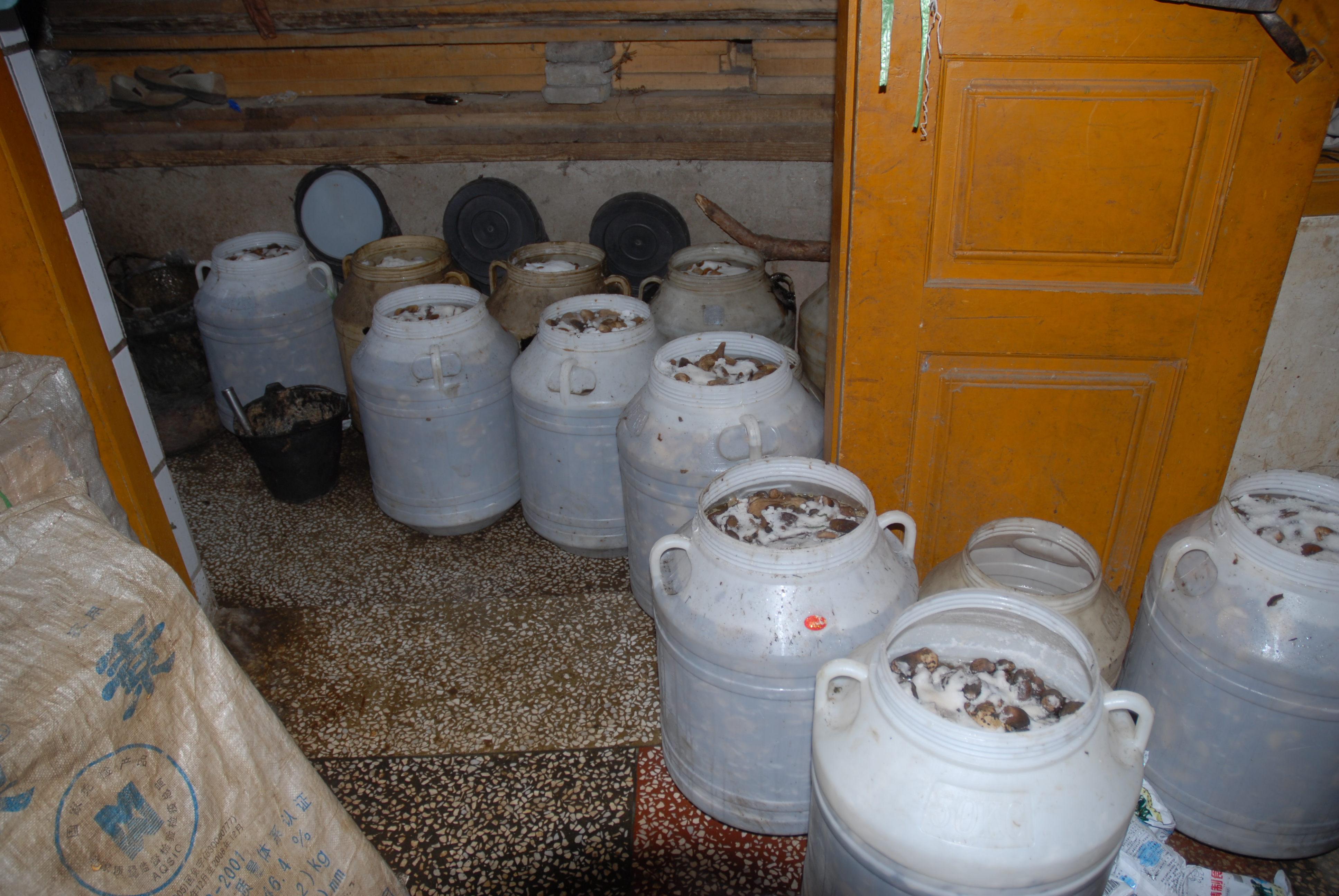


Fig. S9*.* Salting of *Boletus* and other mushrooms in a mushroom merchants shop at Baohe,

Weixi Lisu Autonomous County, Yunnan, China (by Jerzy Falandysz).


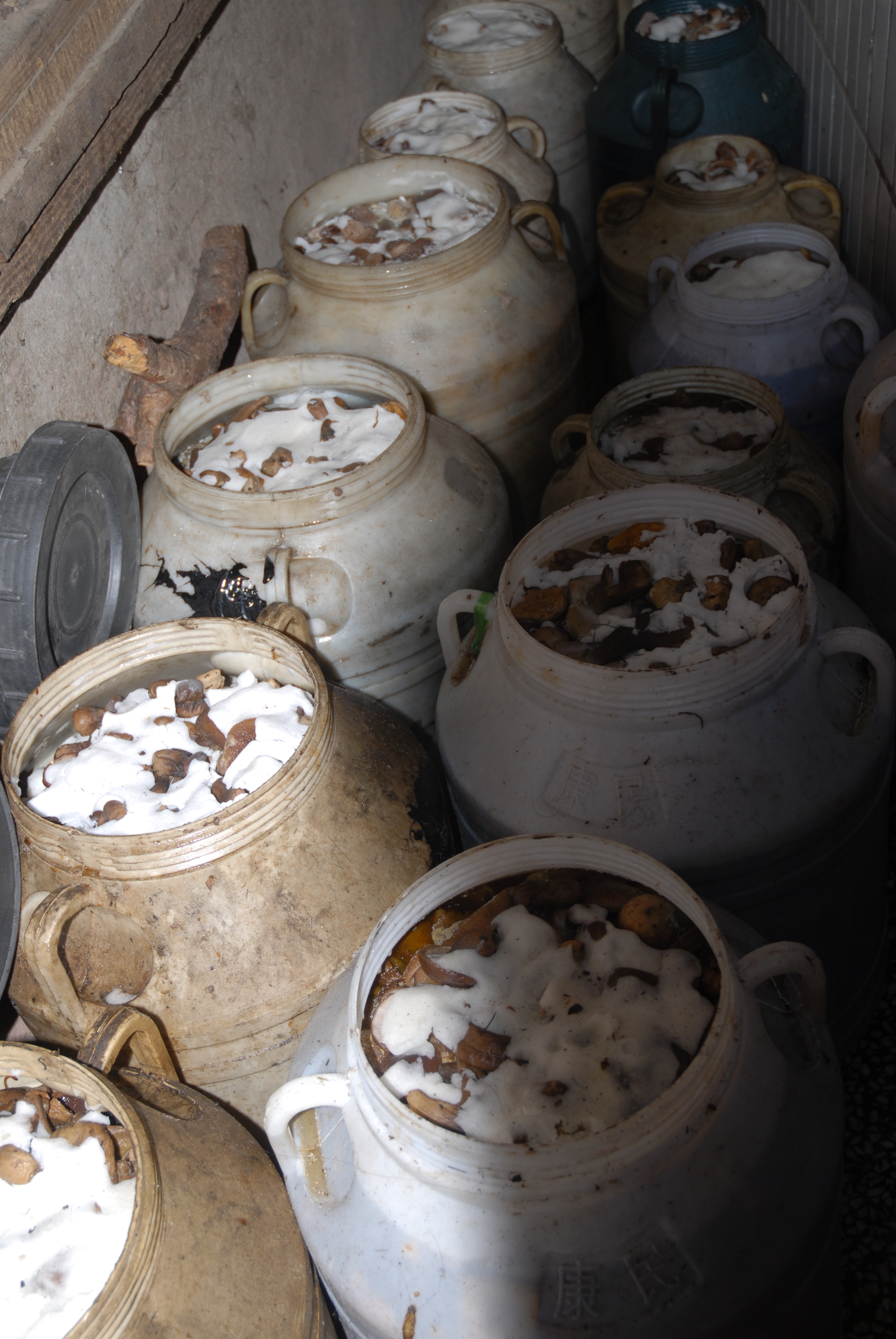


Fig. S10A. Salting of *Boletus* spp. and other mushrooms in a mushroom merchants shop at Baohe, Weixi Lisu Autonomous County, Yunnan, China (by Jerzy Falandysz).
